# SAP30 promotes breast tumor progression by bridging the transcriptional corepressor SIN3 complex and MLL1

**DOI:** 10.1172/JCI168362

**Published:** 2023-09-01

**Authors:** Lei Bao, Ashwani Kumar, Ming Zhu, Yan Peng, Chao Xing, Jennifer E. Wang, Yingfei Wang, Weibo Luo

**Affiliations:** 1 Department of Pathology,; 2 Eugene McDermott Center for Human Growth and Development,; 3 Department of Bioinformatics,; 4 Department of Neurology,; 5 Peter O’Donnell Jr. Brain Institute,; 6 Cecil H. and Ida Green Center for Reproductive Biology Sciences, and; 7 Department of Pharmacology, UT Southwestern Medical Center, Dallas, Texas, USA.

**Keywords:** Oncology, Breast cancer, Epigenetics, Transcription

## Abstract

SAP30 is a core subunit of the transcriptional corepressor SIN3 complex, but little is known about its role in gene regulation and human cancer. Here, we show that SAP30 was a nonmutational oncoprotein upregulated in more than 50% of human breast tumors and correlated with unfavorable outcomes in patients with breast cancer. In various breast cancer mouse models, we found that SAP30 promoted tumor growth and metastasis through its interaction with SIN3A/3B. Surprisingly, the canonical gene silencing role was not essential for SAP30’s tumor-promoting actions. SAP30 enhanced chromatin accessibility and RNA polymerase II occupancy at promoters in breast cancer cells, acting as a coactivator for genes involved in cell motility, angiogenesis, and lymphangiogenesis, thereby driving tumor progression. Notably, SAP30 formed a homodimer with 1 subunit binding to SIN3A and another subunit recruiting MLL1 through specific Phe186/200 residues within its transactivation domain. MLL1 was required for SAP30-mediated transcriptional coactivation and breast tumor progression. Collectively, our findings reveal that SAP30 represents a transcriptional dependency in breast cancer.

## Graphical abstract

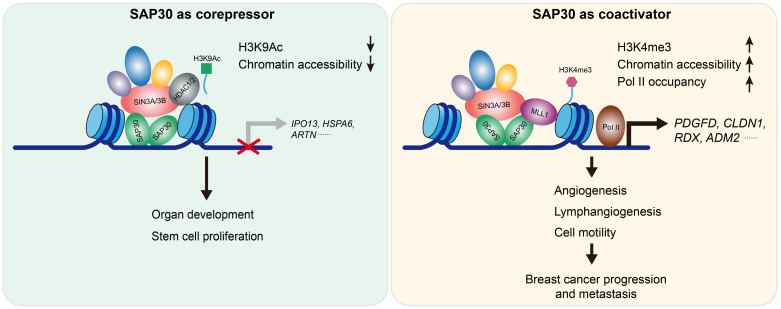

## Introduction

Breast cancer is the most common malignant disease in women, with many patients dying of metastasis. The development of breast cancer toward distant metastasis is a harsh process, wherein the intrinsic regulators confer on cancer cells a substantial advantage for survival. Epigenetic alteration is a notable intrinsic factor because it greatly impacts breast cancer progression and cancer therapy by remodeling the transcriptional landscape (1, 2). Genetic mutations affecting epigenetic factors, including transcription factors, histone modifiers, DNA modifiers, and chromatin remodelers, are frequently observed in breast tumors and can actively disrupt epigenetic signaling at different stages of tumor development, promoting the growth and progression of breast cancer (3–7). Recent studies have appreciated nonmutational epigenetic reprogramming as an emerging scheme of cancer hallmarks (8). For example, c-Myc is highly amplified and drives breast tumorigenesis (9). However, the role and mechanism of transcriptional dependency mediated by nonmutational epigenetic factors in cancer progression are understudied. Understanding nonmutational epigenetic factors may yield an attractive therapeutic vulnerability for the treatment of breast cancer.

SAP30 is a core subunit of the SIN3 protein complex (10, 11), an evolutionarily conserved multisubunit complex involved in development, stem cell pluripotency and self-renewal, cell cycle, senescence, intellectual disability, and tumor progression (12–18). Mammalian SAP30 contains a poorly conserved N-terminal sequence, a zinc finger motif at the central region that may be involved in DNA binding, and a conserved C-terminal SIN3 interaction domain that binds the paired amphipathic helix 3 domain of SIN3A (19, 20). While SIN3A or its paralog SIN3B acts as a scaffold controlling the SIN3 complex assembly through recruitment of the core subunits and their accessory proteins (21), SAP30 also interacts with other complex subunits, including HDAC1, ING1/2, and RBP1, possibly for stabilization of the complex (10, 22, 23). Several studies about SAP30-like protein suggest the involvement of SAP30 in recruitment of the SIN3 complex to the nucleosome and nucleolus (24, 25). The SIN3 protein complex is well known to regulate chromatin compaction and gene silencing through its catalytic subunits HDAC1 and HDAC2 (26, 27). Recent studies, however, reveal predominant enrichment of SIN3A at the promoter (28). SIN3A interacts with TET1 and NANOG to induce pluripotency genes in embryonic stem cells (29, 30). Although SAP30 has been implicated to be essential for the SIN3 complex, its precise role in SIN3 complex, gene regulation, and cancer progression remains poorly understood.

In this study, we demonstrated that SAP30 was upregulated in human breast tumors. SAP30 hijacked the methyltransferase MLL1 to the SIN3 protein complex, which increased trimethyl lysine 4 of histone H3 (H3K4me3), chromatin accessibility, and RNA polymerase II occupancy at the promoters, thereby switching on cancer gene expression and promoting breast tumor development in the mouse models. These findings uncover a noncanonical coactivator function of SAP30 in breast cancer pathogenesis, leading to identification of SAP30 as a possible therapeutic target for the treatment of human breast cancer.

## Results

### SAP30 is upregulated in human breast tumors and correlates with poor survival of patients.

**Figure 1:**
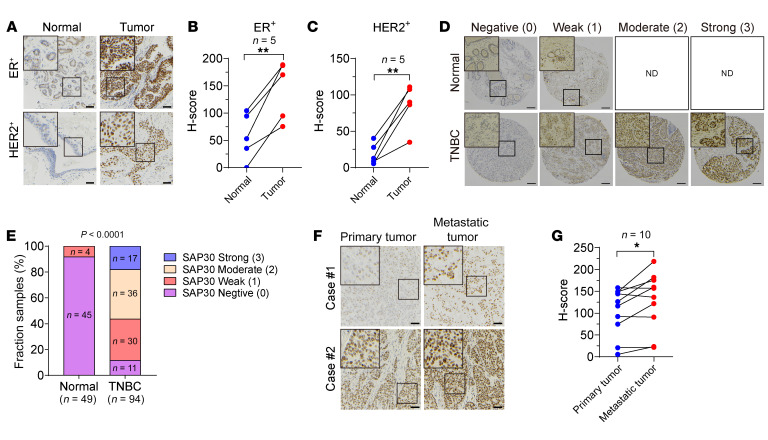
SAP30 is upregulated in human breast tumors. (**A**–**C**) Representative SAP30 immunohistochemical staining in human ER^+^ and HER2^+^ breast tumors and adjacent normal tissues (**A**). Staining is quantified with H-score (**B** and **C**). (**D** and **E**) Representative SAP30 immunohistochemical staining in a human TNBC TMA (**D**). Staining is quantified with intensity score (0–3, **E**). ND, not detected. (**F** and **G**) Representative SAP30 immunohistochemical staining in human paired primary and metastatic breast tumors (**F**). Staining is quantified with H-score (**G**). *P < 0.05, **P < 0.01. Paired 2-tailed Student’s *t* test (**B**, **C**, and **G**); χ^2^ test (**E**). Scale bars: 50 μm (**A** and **F**), 200 μm (**D**).

To study the clinical relevance of the SIN3 complex in human breast cancer, we queried the mRNA expression of the complex core subunits in human breast tumors from several publicly available gene expression data sets. Analysis of The Cancer Genome Atlas (TCGA) breast cancer cohort revealed that breast tumors from more than 50% of patient populations highly expressed *SAP30* and *RBBP7* mRNAs with few other genetic alterations compared with normal breast tissues (Supplemental Figure 1, A and B; supplemental material available online with this article; https://doi.org/10.1172/JCI168362DS1). *SIN3B*, *HDAC1*, *HDAC2*, *SUDS3*, and *RBBP4*, but not *SIN3A* and *SAP18*, were also upregulated to a lesser degree in human breast tumors (Supplemental Figure 1, A and B). However, only *SAP30* mRNA was upregulated in all of 3 Gene Expression Omnibus (GEO) data sets with clinically annotated breast cancer (Supplemental Figure 1, C–E). We further found that mRNAs of *SAP30*, *HDAC1*, *RBBP4*, and *RBBP7* but not other core subunits were significantly induced in all 4 major subtypes of breast tumors (Supplemental Figure 1F). Again, only *SAP30* mRNA exhibited a gradual increase from the lowest level in luminal A breast cancer to the highest level in triple-negative breast cancer (TNBC), which correlated with tumor aggressiveness (Supplemental Figure 1F). Next, we performed immunohistochemical staining to assess SAP30 protein levels in human breast tumors. The specificity of SAP30 antibody was validated in scrambled control (SC) and SAP30-knockout (KO) MDA-MB-231 tumors harvested from the orthotopic xenograft mouse model (Supplemental Figure 1G). SAP30 protein was predominantly localized in the nucleus of cancer cells and upregulated in human ER^+^, HER2^+^, and TNBC tumors compared with their adjacent normal tissues (Figure 1, A–E). Notably, SAP30 protein was significantly elevated in metastatic breast tumors compared with the matched primary tumors (Figure 1, F and G). Kaplan-Meier analysis of 5 independent GEO data sets revealed that high levels of SAP30 were associated with poor overall and metastasis-free survival of patients with breast cancer (Supplemental Figure 1, H–L). Collectively, these findings indicate that SAP30 is upregulated in all major molecular subtypes of breast tumors, particularly metastatic breast tumors, and correlates with poor clinical outcomes in patients with breast cancer.

### SAP30 promotes TNBC growth and distant metastasis in xenograft mouse models.

**Figure 2:**
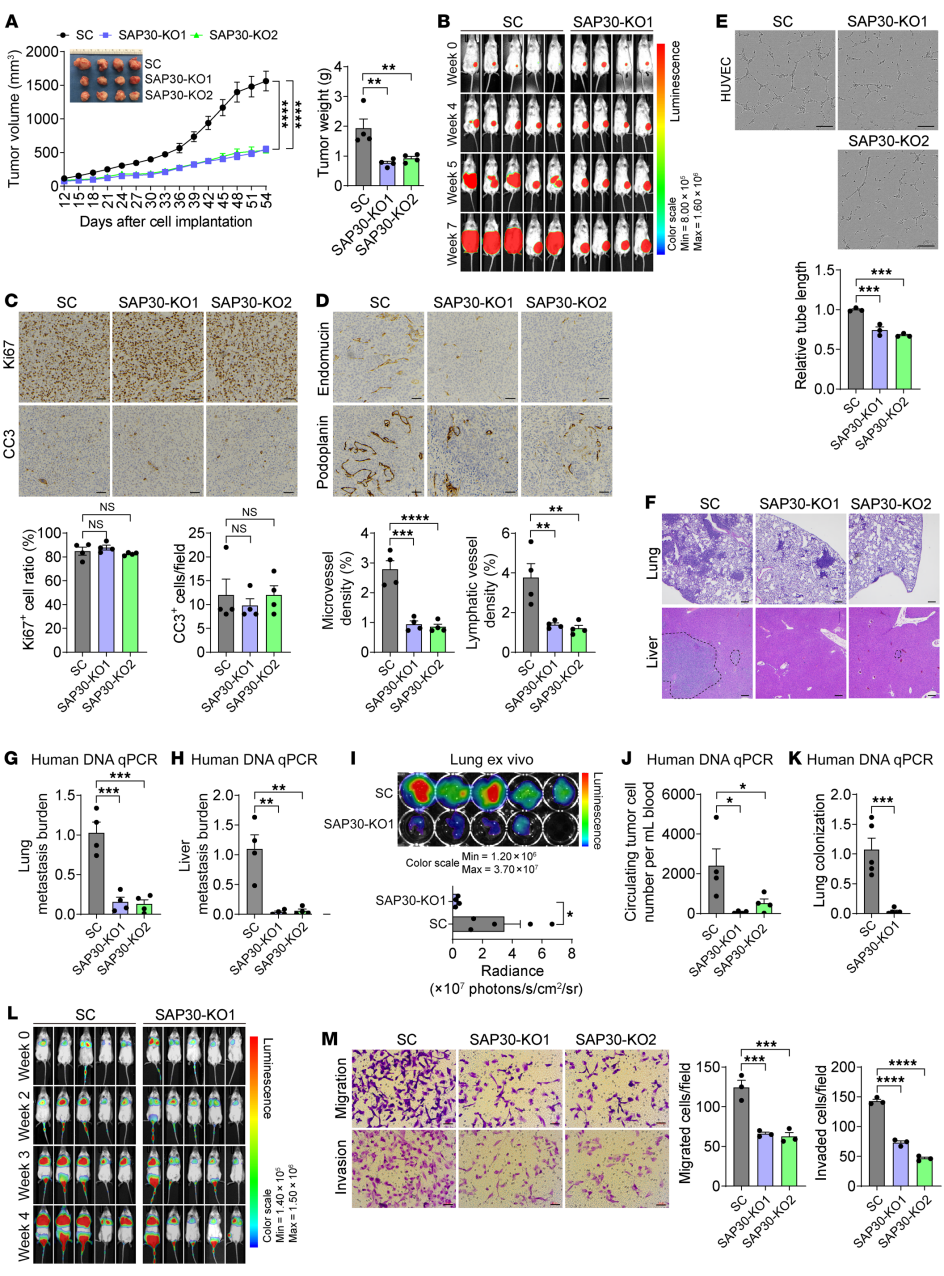
SAP30 promotes TNBC progression in mice. (**A**) Growth of SC and SAP30-KO1 or -KO2 MDA-MB-231 tumors in mice (*n* = 4). After harvesting, tumors were imaged (inset) and weighed (right). (**B**) In vivo bioluminescence imaging in mice after orthotopic implantation of SC or SAP30-KO1 MDA-MB-231 cells (*n* = 4–5). (**C** and **D**) Immunohistochemical staining (top) and quantification (bottom) of Ki67 (**C**), cleaved caspase-3 (CC3) (**C**), endomucin (**D**), and podoplanin (**D**) in SC and SAP30-KO1 or -KO2 MDA-MB-231 tumors (*n* = 4). (**E**) In vitro angiogenesis of HUVECs incubated with conditional media from SC or SAP30-KO1 or -KO2 MDA-MB-231 cells (top). Total tube length is quantified (bottom, *n* = 3). (**F**–**I**) Lung and liver metastasis in mice bearing SC or SAP30-KO1 or -KO2 MDA-MB-231 tumors by H&E staining (**F**, *n* = 4), qPCR (**G** and **H**, *n* = 4), and bioluminescence imaging assays (**I**, *n* = 4–5). (**J**) CTCs in blood from mice bearing SC or SAP30-KO1 or -KO2 MDA-MB-231 tumors by qPCR assay (*n* = 4). (**K** and **L**) Lung colonization of SC and SAP30-KO1 MDA-MB-231 cells by qPCR (**K**) and bioluminescence imaging (**L**) assays (*n* = 5). (**M**) Migration and invasion of SC and SAP30-KO1 or -KO2 MDA-MB-231 cells (left) and their quantification (right). *n* = 3. Data are mean ± SEM. *P < 0.05, **P < 0.01, ***P < 0.001, ****P < 0.0001. Two-tailed Student’s *t* test (**I** and **K**); 1-way ANOVA with Dunnett’s test (**A**, right, **C**–**E**, **G**, **H**, **J**, and **M**); 2-way ANOVA with Dunnett’s test (**A**, left). Scale bars: 50 μm (**C**, **D**, and **M**), 100 μm (**E** and **F**).

To investigate the role of SAP30 in TNBC progression, we knocked out SAP30 without altering other SIN3 complex components in 2 TNBC cell lines, MDA-MB-231 and SUM159, by using 2 independent single guide RNAs (sgRNAs) with the CRISPR/Cas9 technique (Supplemental Figure 2, A and B). SAP30 KO had no effect on proliferation of MDA-MB-231 and SUM159 cells in vitro (Supplemental Figure 2, C and D). Next, we implanted MDA-MB-231-SC, SAP30-KO1, and SAP30-KO2 cells into the mammary fat pad of female NSG mice, respectively. SAP30 KO1 or KO2 significantly inhibited breast tumor growth in mice compared with SC (Figure 2A). These results were confirmed by in vivo bioluminescence imaging (Figure 2B). Similar results were also found in the orthotopic SUM159 xenograft mouse model (Supplemental Figure 2E). To confirm our findings from CRISPR KO studies, we used a short hairpin RNA (shRNA) targeting SAP30 and found that knockdown (Kæ D) of SAP30 similarly attenuated MDA-MB-231 tumor growth in NOD/SCID mice (Supplemental Figure 2, F and G). Collectively, these results indicate that SAP30 promotes TNBC growth in mice.

Immunohistochemical analysis showed that SAP30 KO1 and KO2 had no effect on Ki67 and cleaved caspase-3 levels in MDA-MB-231 tumors (Figure 2C), which excluded an effect on cancer cell proliferation or death as the cause of SAP30-mediated tumor growth. In contrast, the levels of endomucin, a vascular endothelial cell marker, were significantly reduced in SAP30-KO1 and -KO2 tumors compared with SC tumors (Figure 2D). The lymphatic vessel density as shown by podoplanin immunohistochemical staining was also decreased by SAP30 KO1 or KO2 in the peritumoral regions of MDA-MB-231 tumors (Figure 2D). The inhibitory effect of SAP30 KO on density of microvessels and lymphatic vessels was also found in SUM159 tumors (Supplemental Figure 2H). To determine whether tumor angiogenesis is directly regulated by tumor cell–derived SAP30, we performed in vitro angiogenesis assay. Conditional culture media from MDA-MB-231-SC, SAP30-KO1, or SAP30-KO2 cells were collected and incubated with human umbilical vein endothelial cells (HUVECs) for 5 hours. The length of tubes was significantly decreased in HUVECs cultured with SAP30 KO1 or KO2 conditional medium compared with SC conditional medium (Figure 2E). The inhibitory effect on tube formation was also found in HUVECs cultured in SUM159–SAP30 KO1 or KO2 conditional medium (Supplemental Figure 2I). Together, these in vitro and in vivo results indicate that SAP30 increases angiogenesis and lymphangiogenesis in TNBC.

Extensive spontaneous metastasis to the lungs, lymph nodes, and/or liver was detected by H&E staining in mice bearing MDA-MB-231-SC or SUM159-SC tumors, which was significantly inhibited by SAP30 KO or KD (Figure 2F and Supplemental Figure 2, J and K). These metastasis burden results were further confirmed by quantitative PCR (qPCR) quantification of human genomic DNA in mouse lungs and liver and ex vivo bioluminescence imaging of mouse lungs (Figure 2, G–I, and Supplemental Figure 2, L and M). To rule out the possibility that reduced metastatic burden in mice bearing SAP30-KO or -KD tumors is due to the reduced primary tumor volume, we euthanized SAP30-KO tumor–bearing mice 15 days later than control mice when SAP30-KO tumor volumes matched with those of SC tumors in mice at day 48 (Supplemental Figure 2N). Again, few metastatic foci were found in the lungs from mice with the matched volume of SAP30-KO1 tumors (Supplemental Figure 2, O and P). Next, we studied the effect of SAP30 on circulating tumor cells (CTCs) in the orthotopic xenograft mouse model and found that CTCs were markedly eliminated in blood from mice bearing SAP30-KO1 or -KO2 tumors (Figure 2J). Finally, we studied whether SAP30 controls TNBC cell colonization at distant organs, a critical step for metastatic tumor outgrowth. SC, SAP30-KO1, or SAP30-KD MDA-MB-231 cells were injected into the tail vein of female NOD/SCID mice. Three weeks after injection, mouse lungs were harvested for detection of human genomic DNA with qPCR. As expected, a fair amount of human genomic DNA was detected in the lungs of mice injected with SC cells, indicating cancer cell colonization and outgrowth in the lungs (Figure 2K and Supplemental Figure 2Q). In contrast, few SAP30-KO1 or -KD MDA-MB-231 cells were detected in mouse lungs (Figure 2K and Supplemental Figure 2Q). These results were confirmed by in vivo bioluminescence imaging (Figure 2L and Supplemental Figure 2, R and S). Consistent with in vivo metastasis results, in vitro Boyden chamber assay showed that SAP30 KO1 or KO2 markedly reduced migration and invasion of MDA-MB-231 and SUM159 cells (Figure 2M and Supplemental Figure 2T). Collectively, these results indicate that SAP30 promotes distant TNBC metastasis by controlling multiple metastatic cascades in mice.

### SAP30 promotes luminal mammary tumor initiation, growth, and distant metastasis in a genetically modified mouse model.

**Figure 3:**
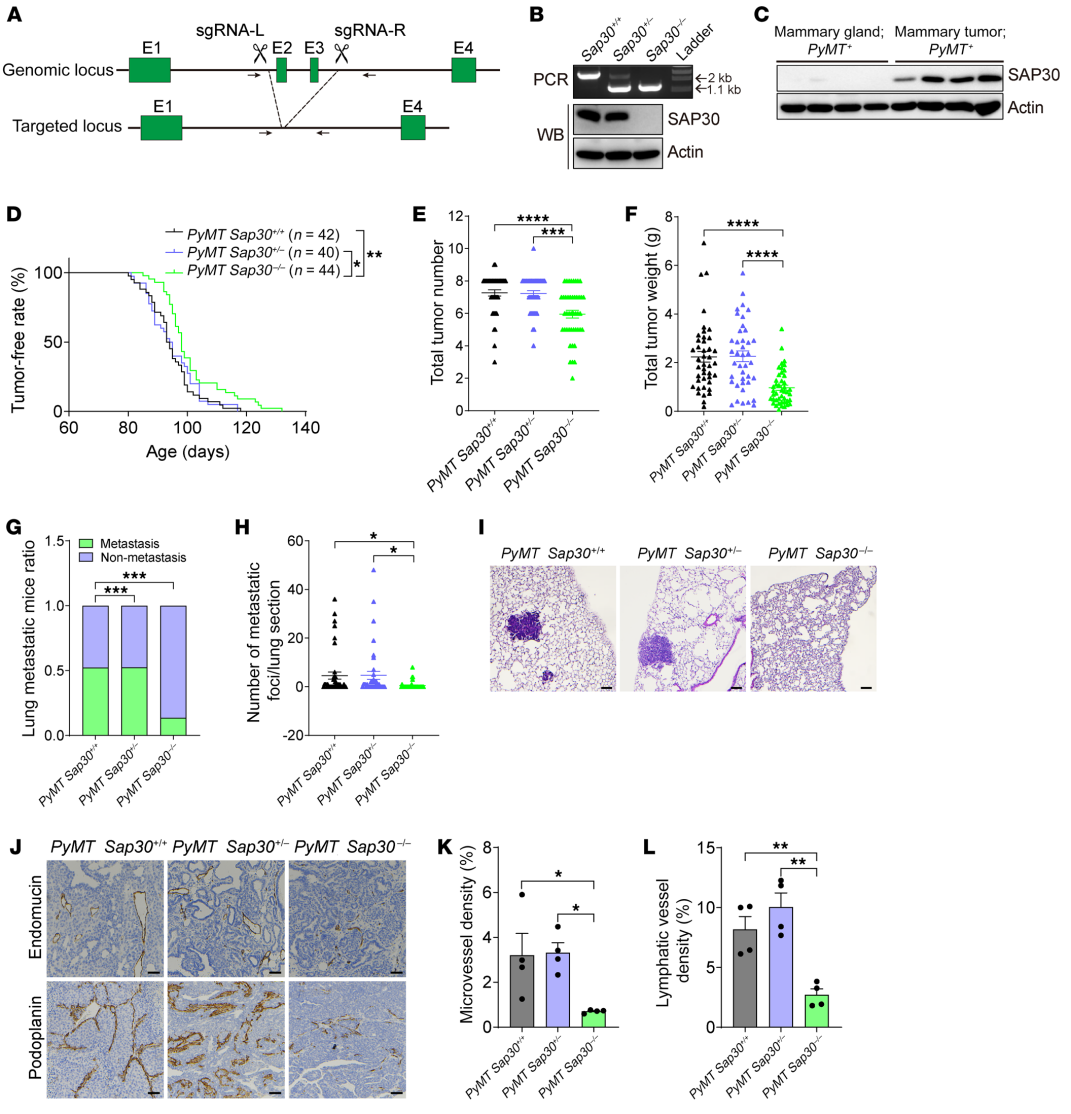
Loss of *Sap30* suppresses mouse mammary tumor initiation and progression. (**A**) Scheme of constitutive *Sap30* KO strategy in mice. Exons and introns are not drawn to scale. (**B**) PCR genotyping and Western blotting (WB) of SAP30 protein in *Sap30^+/+^*, *Sap30^+/–^*, and *Sap30^–/–^* mice. (**C**) Immunoblot of SAP30 and actin proteins in normal mammary gland and *MMTV-PyMT* mammary tumors from mice. (**D**) Tumor-free period in *MMTV*-*PyMT Sap30^+/+^*, *MMTV*-*PyMT Sap30^+/–^*, and *MMTV*-*PyMT* *Sap30^–/–^* mice. (**E** and **F**) Mammary tumor number (**E**) and weight (**F**) in *MMTV*-*PyMT Sap30^+/+^*, *MMTV*-*PyMT Sap30^+/–^*, and *MMTV*-*PyMT* *Sap30^–/–^* mice. (**G**) Lung metastasis ratio in *MMTV*-*PyMT Sap30^+/+^*, *MMTV*-*PyMT Sap30^+/–^*, and *MMTV*-*PyMT* *Sap30^–/–^* mice. (**H** and **I**) Quantification of metastatic foci (**H**) from lung H&E images (**I**) in *MMTV*-*PyMT Sap30^+/+^*, *MMTV*-*PyMT Sap30^+/–^*, and *MMTV*-*PyMT* *Sap30^–/–^* mice. (**J**–**L**) Immunohistochemical staining of endomucin and podoplanin in *MMTV*-*PyMT Sap30^+/+^*, *MMTV*-*PyMT Sap30^+/–^*, and *MMTV*-*PyMT* *Sap30^–/–^* mammary tumors (**J**). Endomucin-positive (**K**) and podoplanin-positive (**L**) tumor areas are quantified (mean ± SEM, *n* = 4). *P < 0.05, **P < 0.01, ***P < 0.001, ****P < 0.0001. Log-rank (Mantel-Cox) test (**D**); χ^2^ test (**G**); 1-way ANOVA with Tukey’s test (**E**, **F**, **H**, **K**, and **L**). Scale bars: 100 μm (**I**), 50 μm (**J**).

To determine the role of SAP30 in luminal breast cancer development, we generated *Sap30*-KO mice by deleting exons 2 and 3 of the *Sap30* gene with the CRISPR/Cas9 technique (Figure 3A) and crossed these mice with MMTV-PyMT transgenic mice, a well-characterized mouse model of luminal mammary tumor. Homozygous but not heterozygous deletion of *Sap30* completely knocked out SAP30 protein in mammary glands (Figure 3B). In line with human breast tumors (Figure 1, A–E), MMTV-PyMT tumors strongly expressed SAP30 protein compared with normal mammary glands (Figure 3C). Homozygous KO of SAP30 modestly but significantly inhibited mammary tumor initiation compared with wild-type (WT) or heterozygous deletion of SAP30 (Figure 3D). At postnatal day 155, we harvested mammary tumors from these mouse models and found that both total tumor number and weight were significantly reduced in SAP30-homozygous-KO mice compared with SAP30-WT or heterozygous mice (Figure 3, E and F). Likewise, spontaneous lung metastasis was significantly inhibited in SAP30-homozygous-KO mice as shown by the percentage of mice with lung metastasis and the number of lung metastatic foci (Figure 3, G–I). Immunohistochemical staining with anti-endomucin and anti-podoplanin antibodies showed reduced tumor angiogenesis and lymphangiogenesis, respectively, in SAP30-KO mice (Figure 3, J–L). Taken together, these results indicate that SAP30 promotes luminal breast tumor initiation and progression in a genetically modified mouse model.

### SAP30 promotes breast cancer progression in a SIN3A/3B-dependent manner.

**Figure 4:**
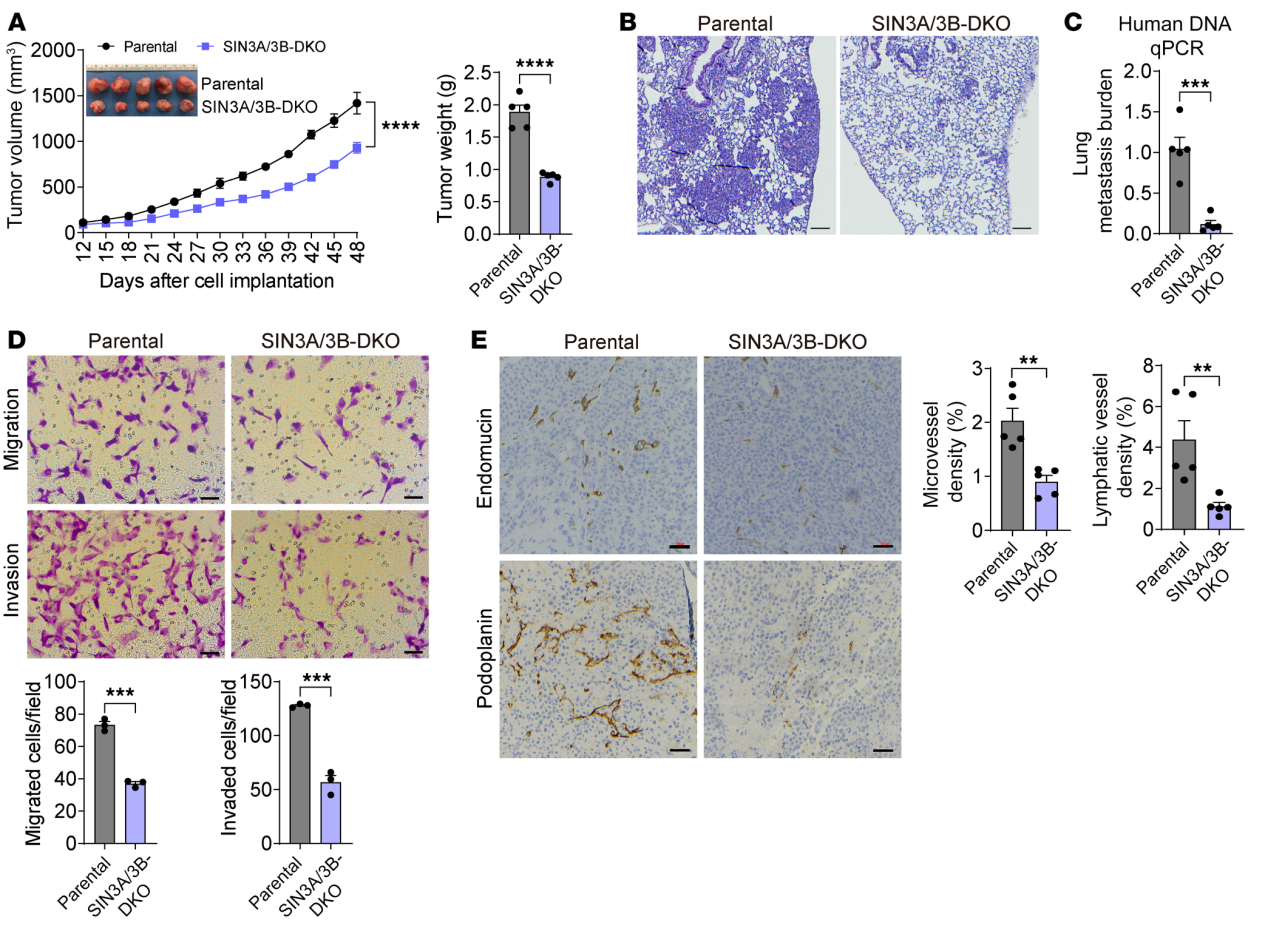
The SIN3 complex promotes TNBC progression in mice. (**A**) Growth of parental and SIN3A/3B-DKO MDA-MB-231 tumors in mice (*n* = 5). After harvesting, tumors were imaged (inset) and weighed (right). (**B** and **C**) Lung metastasis in mice bearing parental or SIN3A/3B-DKO MDA-MB-231 tumors by H&E staining (**B**) and qPCR assay (**C**). *n* = 5. (**D**) Migration and invasion of parental and SIN3A/3B-DKO MDA-MB-231 cells (top). Migrated or invaded cell numbers are quantified (bottom). *n* = 3. (**E**) Immunohistochemical staining of endomucin and podoplanin in parental and SIN3A/3B-DKO MDA-MB-231 tumors (left). Endomucin- and podoplanin-positive tumor areas are quantified (right). *n* = 5. Data are mean ± SEM. **P < 0.01, ***P < 0.001, ****P < 0.0001. Two-tailed Student’s *t* test (**A**, right, and **C**–**E**); 2-way ANOVA with Šidák’s test (**A**, left). Scale bars: 100 μm (**B**), 50 μm (**D** and **E**).

To determine whether the SIN3 complex mediates breast cancer progression, we knocked out both SIN3A and SIN3B proteins with the CRISPR/Cas9 technique in MDA-MB-231 cells (Supplemental Figure 3A). The orthotopic mouse xenograft studies showed that SIN3A/3B double KO (DKO) significantly reduced MDA-MB-231 tumor growth and lung metastasis in mice (Figure 4, A–C). Migration and invasion of MDA-MB-231 cells were significantly inhibited by SIN3A/3B DKO in vitro (Figure 4D). Endomucin and podoplanin immunohistochemical staining revealed reduced angiogenesis and lymphangiogenesis, respectively, in SIN3A/3B-DKO tumors compared with parental MDA-MB-231 tumors (Figure 4E). In vitro angiogenesis assay supported that SIN3A/3B DKO in tumor cells resulted in inhibition of HUVEC tube formation (Supplemental Figure 3B). Collectively, these findings indicate that SIN3A and SIN3B phenocopy SAP30 to promote breast tumor angiogenesis, growth, and metastasis in vitro and in vivo.

**Figure 5:**
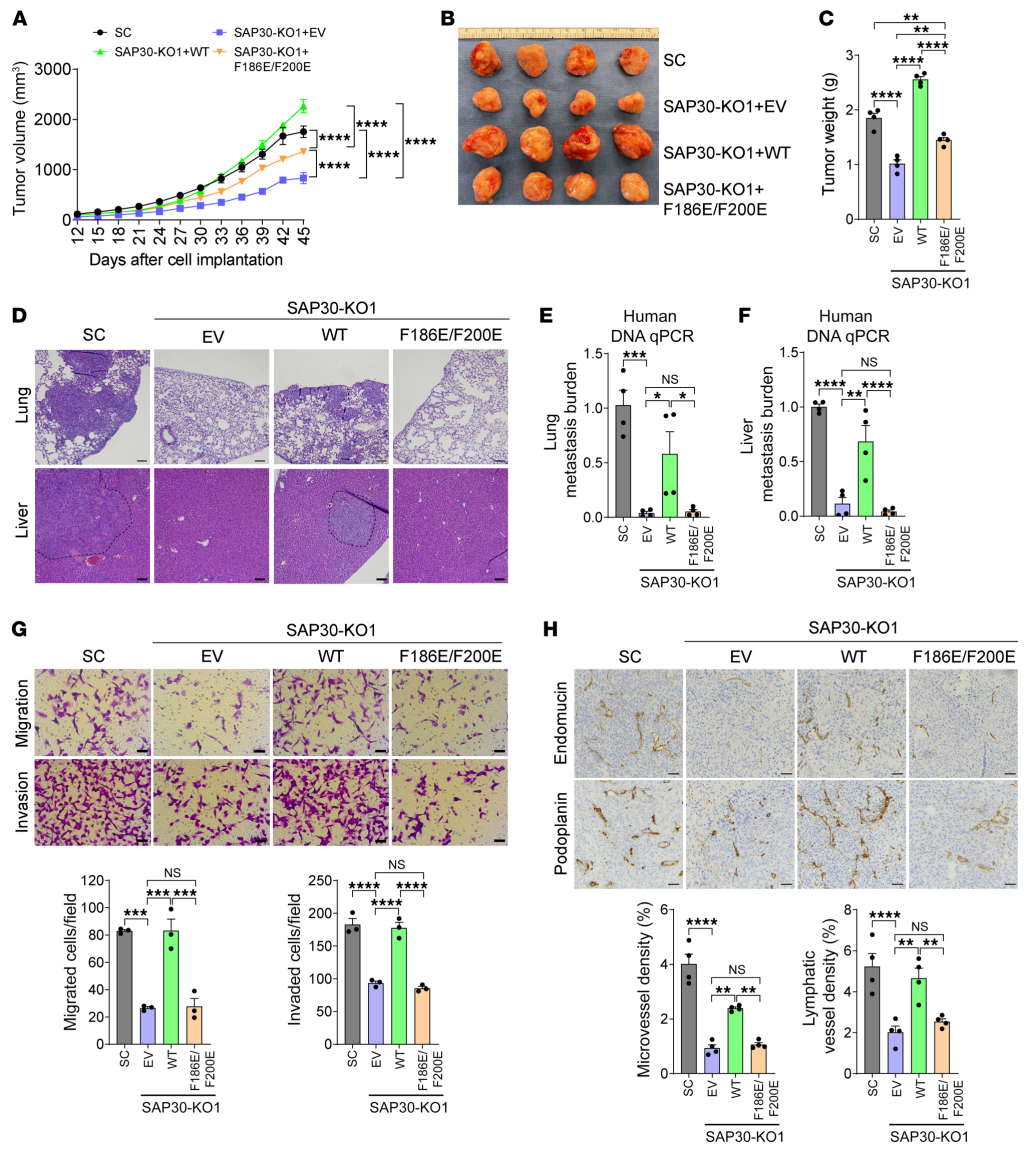
SAP30 promotes breast cancer progression in a SIN3A/3B-dependent manner. (**A**–**C**) Growth of SC, SAP30-KO, and SAP30-rescue MDA-MB-231 tumors in mice (**A**). After harvesting, tumors were imaged (**B**) and weighed (**C**). *n* = 4. (**D**–**F**) Lung and liver metastasis in mice bearing SC, SAP30-KO, or SAP30-rescue MDA-MB-231 tumors by H&E staining (**D**) and qPCR assay (**E** and **F**). *n* = 4. (**G**) Migration and invasion of SC, SAP30-KO, and SAP30-rescue MDA-MB-231 cells (top). Migrated or invaded cell numbers are quantified (bottom). *n* = 3. (**H**) Immunohistochemical staining of endomucin and podoplanin in SC, SAP30-KO, and SAP30-rescue MDA-MB-231 tumors (top). Endomucin- and podoplanin-positive tumor areas are quantified (bottom). *n* = 4. Data are mean ± SEM. *P < 0.05, **P < 0.01, ***P < 0.001, ****P < 0.0001. One-way ANOVA with Tukey’s test (**C** and **E**–**H**); 2-way ANOVA with Tukey’s test (**A**). Scale bars: 100 μm (**D**), 50 μm (**G** and **H**).

We next studied whether SAP30 mediates breast cancer progression through the SIN3 complex. A previous structural study reported that SAP30 binds to SIN3A mainly through its amino acid (aa) residues Val148, Phe186, and Phe200 (20). We generated the rescued cell lines by transducing MDA-MB-231 SAP30-KO cells with lentivirus encoding empty vector (EV), WT SAP30, or F186E/F200E mutant SAP30 (Supplemental Figure 3C). Coimmunoprecipitation (co-IP) assay confirmed that F186E/F200E abolished association of SAP30 with the SIN3 complex in MDA-MB-231 cells (Supplemental Figure 3D). Next, we orthotopically implanted SAP30-KO and rescue cell lines and MDA-MB-231-SC cells into the mammary fat pad of female NSG mice, respectively. As expected, SAP30 KO blocked MDA-MB-231 tumor growth and distant metastasis to the lungs and liver, which was fully rescued by re-expression of WT SAP30 (Figure 5, A–F). Expression of F186E/F200E mutant prevented the rescued effect of SAP30 on tumor growth and metastasis to the lungs and liver, although it slightly increased SAP30-KO tumor growth, maybe owing to its very high protein levels (Figure 5, A–F, and Supplemental Figure 3C). Consistent with their effect on distant metastasis, WT but not F186E/F200E SAP30 restored migration and invasion of MDA-MB-231 SAP30-KO1 cells in vitro (Figure 5G). Likewise, reduced tumor angiogenesis and lymphangiogenesis in MDA-MB-231 SAP30-KO1 tumors were also reversed by WT but not F186E/F200E SAP30 (Figure 5H). A similar rescued effect on angiogenesis was also observed in HUVECs in vitro (Supplemental Figure 3E). Collectively, these findings indicate that SAP30 promotes breast tumor growth, tumor angiogenesis, lymphangiogenesis, and distant metastasis in a SIN3A/3B-dependent manner.

### The canonical SIN3 corepressor complex is dispensable for breast cancer progression.

**Figure 6:**
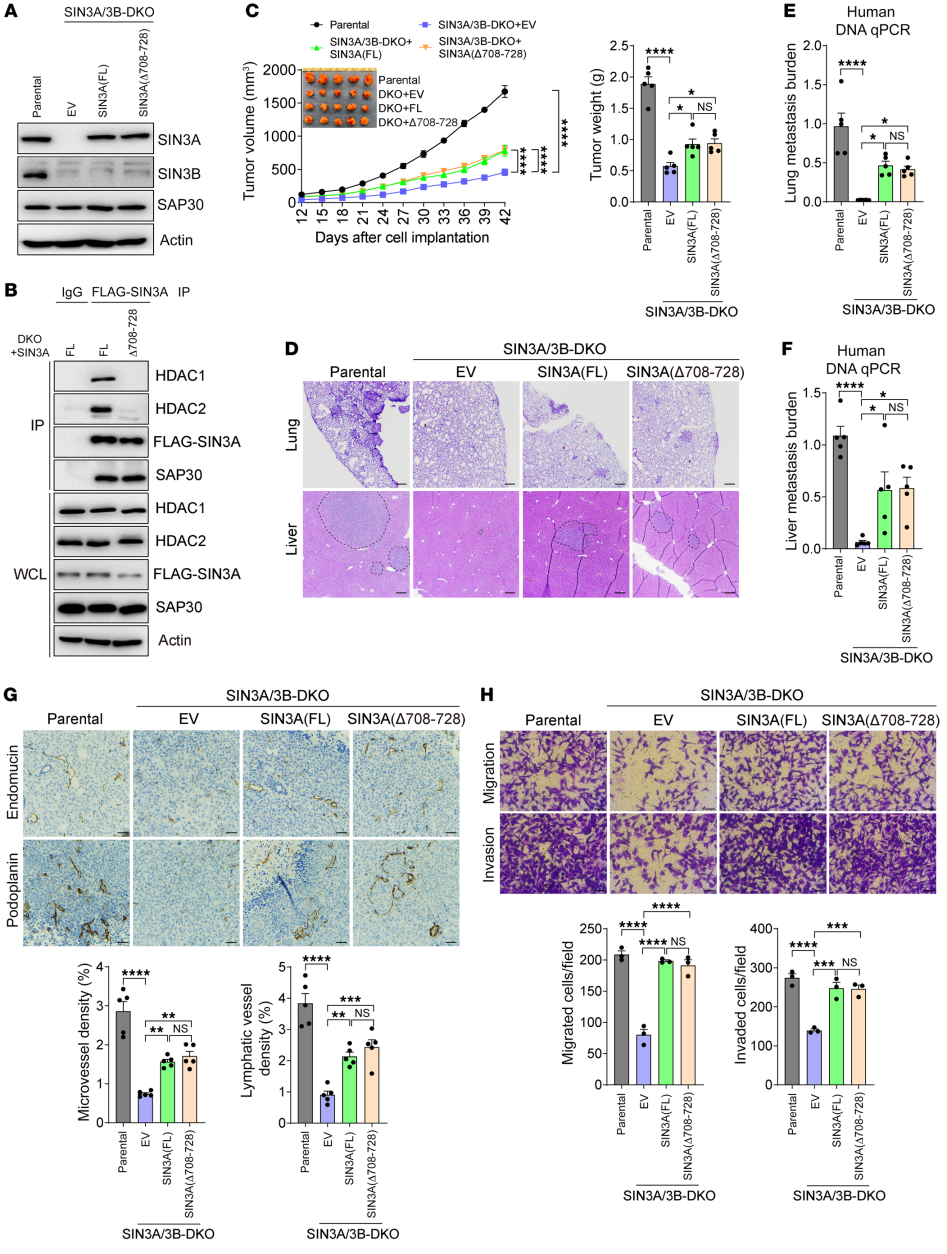
Canonical SIN3 complex is dispensable for breast cancer progression in mice. (**A**) Immunoblot of indicated proteins in parental, SIN3A/3B-DKO, and SIN3A-rescue MDA-MB-231 cells. FL, full-length. SIN3A and SIN3B blots were run in parallel using the same biological samples. (**B**) Co-IP assay showing that Δ708–728 mutation abolishes FLAG-SIN3A binding to HDAC1 and HDAC2, but not SAP30, in SIN3A/3B-DKO MDA-MB-231 cells (*n* = 2). WCL, whole-cell lysate. (**C**) Growth of parental, SIN3A/3B-DKO, and SIN3A-rescue MDA-MB-231 tumors in mice (*n* = 5). After harvesting, tumors were imaged (inset) and weighed (right). (**D**–**F**) Lung and liver metastasis in mice bearing parental, SIN3A/3B-DKO, or SIN3A-rescue MDA-MB-231 tumors by H&E staining (**D**) and qPCR assay (**E** and **F**). *n* = 5. (**G**) Immunohistochemical staining of endomucin and podoplanin in parental, SIN3A/3B-DKO, and SIN3A-rescue MDA-MB-231 tumors (top). Endomucin- and podoplanin-positive tumor areas are quantified (bottom). *n* = 5. (**H**) Migration and invasion of parental, SIN3A/3B-DKO, and SIN3A-rescue MDA-MB-231 cells (top). Migrated or invaded cell numbers are quantified (bottom). *n* = 3. Data are mean ± SEM. *P < 0.05, **P < 0.01, ***P < 0.001, ****P < 0.0001. One-way ANOVA with Tukey’s test (**C**, right, and **E**–**H**); 2-way ANOVA with Tukey’s test (**C**, left). Scale bars: 100 μm (**D**), 50 μm (**G** and **H**).

We next determined whether the corepressor function of the SIN3 complex controls breast cancer progression. To this end, we first mapped the SIN3A domain binding to HDAC1/2 using a series of SIN3A protein truncates. Co-IP assay showed that deletion of amino acids 687–829 abolished the interaction of SIN3A with HDAC1 and HDAC2 in transfected HEK293T cells (Supplemental Figure 4A), which validated a previous report (31). However, this deletion mutant impaired SAP30 binding to SIN3A in HEK293T cells (Supplemental Figure 4A). Further analysis with a series of SIN3A deletion mutants narrowed down the HDAC1 and HDAC2 binding site to amino acids 709–728 of SIN3A in HEK293T cells (Supplemental Figure 4, B and C). We next reintroduced full-length (FL) SIN3A and its aa708–728 deletion mutant into MDA-MB-231 SIN3A/3B-DKO cells and confirmed that deletion of amino acids 708–728 abolished binding of both HDAC1 and HDAC2 to SIN3A without interfering with SIN3A-SAP30 interaction in MDA-MB-231 cells (Figure 6, A and B). We implanted parental and SIN3A/3B-DKO MDA-MB-231 cells and FL SIN3A or SIN3A (Δ708–728) deletion mutant rescue cells, respectively, into the mammary fat pad of female NSG mice, and found that single expression of FL SIN3A partially reversed the inhibitory effect of SIN3A/3B DKO on tumor growth, angiogenesis, lymphangiogenesis, and distant metastasis to lungs and liver in mice (Figure 6, C–G). Remarkably, the identical rescued effects were observed for SIN3A (Δ708–728) in mice (Figure 6, C–G). Likewise, FL SIN3A and its Δ708–728 deletion mutant equally and significantly restored the ability of migration and invasion of SIN3A/3B-DKO MDA-MB-231 cells in vitro (Figure 6H). Taken together, these findings indicate that HDAC1/2-mediated SIN3/SAP30 corepressor function is not essential for breast cancer progression.

### SAP30 coactivates genes involved in angiogenesis, lymphangiogenesis, and cell motility.

**Figure 7:**
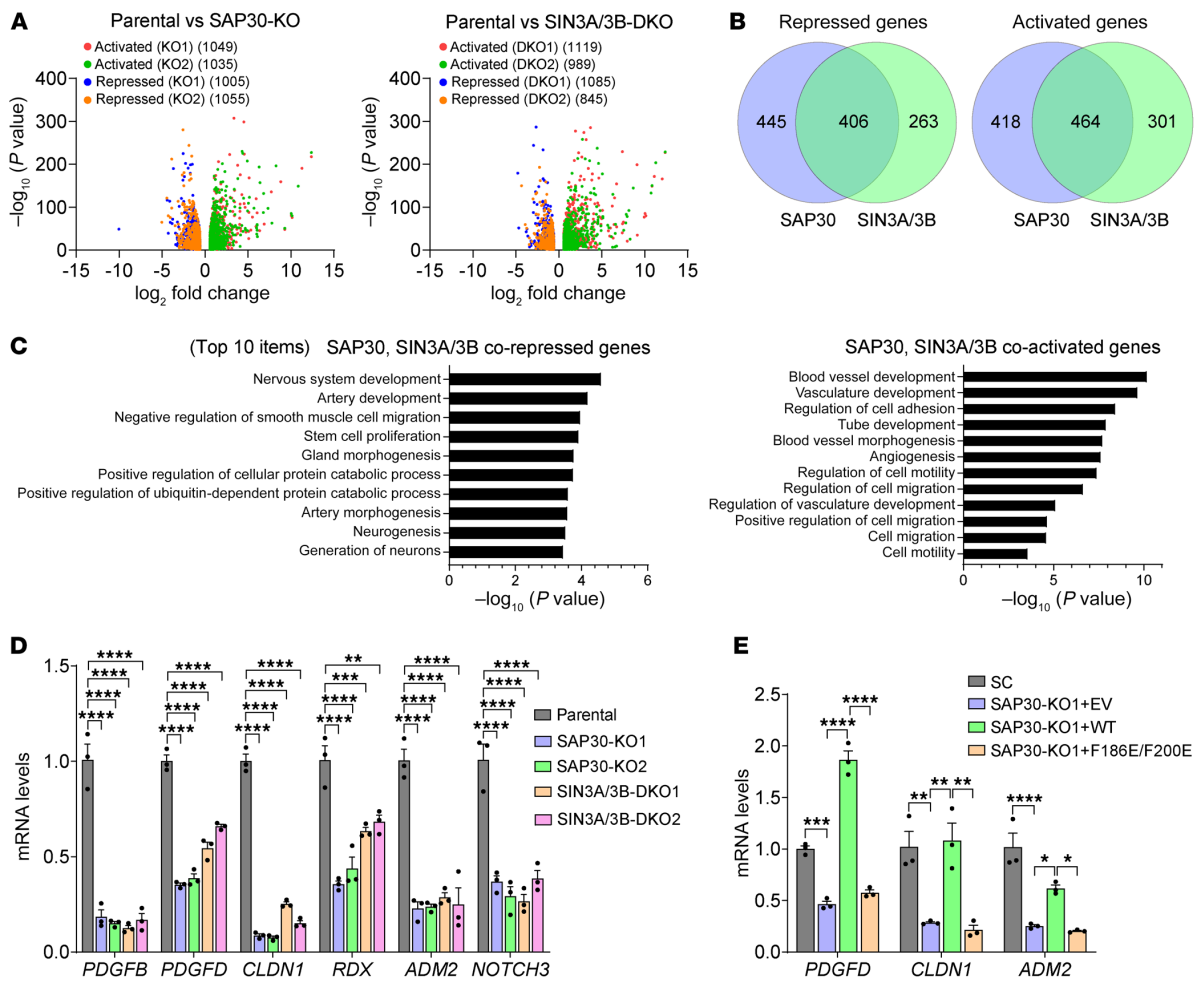
SAP30 coactivates genes involved in cell motility, angiogenesis, and lymphangiogenesis in breast cancer cells. (**A**) Volcano plot of SAP30 (left) and SIN3A/3B (right) target genes in MDA-MB-231 cells (*n* = 2). (**B**) Venn diagram of overlapped genes regulated by SAP30 and SIN3A/3B in MDA-MB-231 cells (*n* = 2). (**C**) Gene ontology analysis of SAP30- and SIN3A/3B-coregulated genes in MDA-MB-231 cells (*n* = 2) . (**D**) RT-qPCR analysis of indicated mRNAs in parental, SAP30-KO, and SIN3A/3B-DKO MDA-MB-231 cells (*n* = 3). (**E**) RT-qPCR analysis of indicated mRNAs in SC, SAP30-KO, and SAP30-rescue MDA-MB-231 cells (*n* = 3). Data are mean ± SEM. *P < 0.05, **P < 0.01, ***P < 0.001, ****P < 0.0001. One-way ANOVA with Dunnett’s test (**D**) or Tukey’s test (**E**).

Given the identical role of SAP30 in both luminal breast cancer and TNBC (Figures 2 and 3), we used TNBC models to investigate the mechanism of SAP30-mediated breast tumor progression. We analyzed SAP30- and/or SIN3A/3B-dependent transcriptome in MDA-MB-231 cells with RNA sequencing (RNA-Seq). SAP30 KO1 or KO2 altered an almost equal number of upregulated and downregulated genes in MDA-MB-231 cells (Figure 7A, left). A similar pattern of gene regulation was observed in SIN3A/3B-DKO cells (Figure 7A, right). Interestingly, about half of SAP30 target genes overlapped with SIN3A/3B target genes (Figure 7B). Gene ontology analysis of these overlapped genes revealed that SIN3A/3B/SAP30-corepressed genes were involved in development of multiple organs, which was consistent with previous findings (13); whereas angiogenesis- and cell migration–related pathways were highly enriched among SIN3A/3B/SAP30-coactivated genes (Figure 7C), which supported SAP30/SIN3A/3B-mediated tumor phenotypes in breast cancer mouse models (Figures 2–4). Reverse transcription quantitative PCR (RT-qPCR) assay validated that SAP30 KO or SIN3A/3B DKO similarly decreased the expression of 6 representative genes, *PDGFB*, *PDGFD*, *CLDN1*, *RDX*, *ADM2*, and *NOTCH3*, which were selected based on their well-known role in angiogenesis and/or cell migration, in MDA-MB-231 and SUM159 cells (Figure 7D and Supplemental Figure 5, A and B). Inhibition of PDGFD by SAP30 KO was confirmed at the protein level (Supplemental Figure 5C). Moreover, WT but not F186E/F200E SAP30 was able to restore the expression of *PDGFD*, *CLDN1*, and *ADM2* in MDA-MB-231 SAP30-KO1 cells (Figure 7E). Together, these results indicate that SAP30 coactivates genes involved in angiogenesis, lymphangiogenesis, and cell motility in a SIN3A/3B-dependent manner.

**Figure 8:**
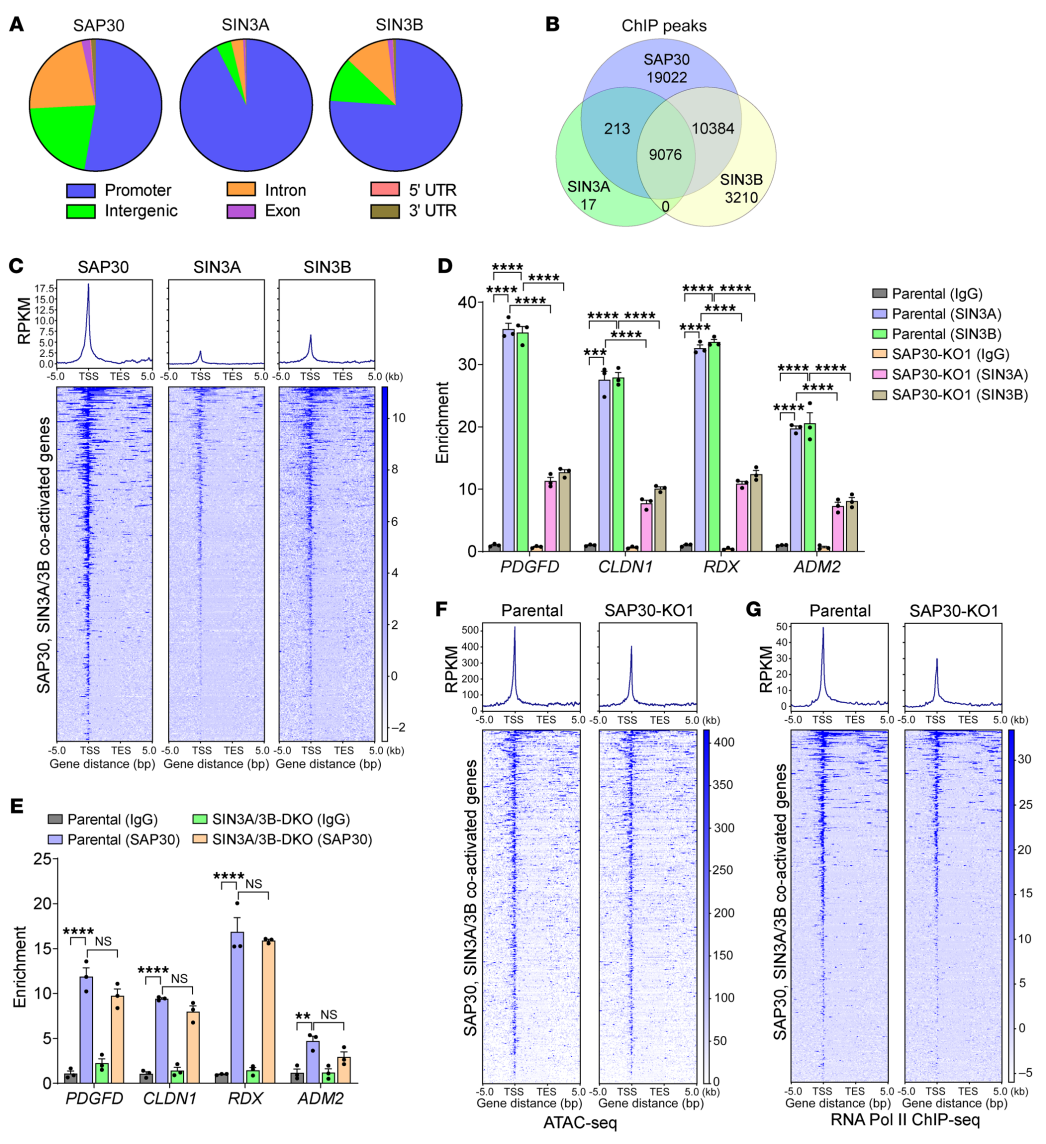
SAP30 recruits the SIN3 complex to the chromatin and regulates chromatin accessibility in breast cancer cells. (**A**) Genomic distribution analysis of SAP30, SIN3A, and SIN3B in MDA-MB-231 cells (*n* = 2). (**B**) Venn diagram of overlapped ChIP-Seq peaks by SAP30, SIN3A, and SIN3B (*n* = 2). (**C**) Metagene plot and heatmap of ChIP-Seq assay showing occupancies of SAP30, SIN3A, and SIN3B on their coactivated genes (*n* = 2). RPKM, reads per kilobase per million mapped reads; TSS, transcription start site; TES, transcription end site. (**D**) ChIP-qPCR assay showing relative SIN3A and SIN3B occupancies on representative SAP30-, SIN3A/3B-coactivated genes in parental and SAP30-KO1 MDA-MB-231 cells (*n* = 3). (**E**) ChIP-qPCR assay showing relative SAP30 occupancy on representative SAP30-, SIN3A/3B-coactivated genes in parental and SIN3A/3B-DKO MDA-MB-231 cells (*n* = 3). (**F**) Metagene plot and heatmap of ATAC-seq assay showing chromatin accessibility on SAP30-, SIN3A/3B-coactivated genes in parental and SAP30-KO1 MDA-MB-231 cells (*n* = 3). (**G**) Metagene plot and heatmap of ChIP-Seq assay showing RNA polymerase II occupancy on SAP30-, SIN3A/3B-coactivated genes in parental and SAP30-KO1 MDA-MB-231 cells (*n* = 2). Data are mean ± SEM. **P < 0.01, ***P < 0.001, ****P < 0.0001. Two-way ANOVA with Tukey’s test (**D** and **E**).

We next performed chromatin immunoprecipitation sequencing (ChIP-Seq) in MDA-MB-231 cells to study whether SAP30 directly controls the transcription of genes involved in angiogenesis and cell motility. SAP30 was highly enriched at the promoter, intergenic region, and intron (Figure 8A). SIN3A and SIN3B predominantly occupied the promoter, although a quarter of SIN3B was also enriched at the intergenic region and intron (Figure 8A). Remarkably, the majority of SIN3A ChIP-Seq peaks and 50% of SAP30 ChIP-Seq peaks overlapped and positively correlated in MDA-MB-231 cells (Figure 8B and Supplemental Figure 5D). SIN3B ChIP-Seq peaks also overlapped and correlated with SAP30 ChIP-Seq peaks to a lesser degree (Figure 8B and Supplemental Figure 5E). As expected, we detected co-occupancy of SAP30, SIN3A, and SIN3B at the transcription start sites of corepressed genes (Supplemental Figure 5, F–H). A similar enrichment pattern was also found at their coactivated genes (Figure 8C and Supplemental Figure 5, I and J). Enrichment of SAP30, SIN3A, and SIN3B at the coactivated genes *PDGFD*, *CLDN1*, *RDX*, and *ADM2* was validated by ChIP-qPCR in MDA-MB-231 cells (Figure 8D). Notably, SAP30 KO significantly reduced occupancy of SIN3A and SIN3B at these coactivated genes (Figure 8D). In contrast, SIN3A/3B DKO had no effect on SAP30 binding to coactivated genes in MDA-MB-231 cells (Figure 8E). These results indicate that SAP30 mediates the recruitment of SIN3A/3B to their coactivated genes in breast cancer cells.

The catalytic subunits HDAC1 and HDAC2 are responsible for SIN3 complex–mediated gene silencing by deacetylating histones (26, 27). Indeed, we found that SAP30 KO1 decreased HDAC2 enrichment at SAP30/SIN3A/3B-corepressed genes in MDA-MB-231 cells (Supplemental Figure 5K). In line with this, SAP30 KO1 increased H3K9ac enrichment at these corepressed genes (Supplemental Figure 5, G, H, and L). However, HDAC2 and H3K9ac enrichment at SAP30/SIN3A/3B-coactivated genes was not altered in SAP30-KO1 cells (Supplemental Figure 5, M and N). These results suggest that an unknown SIN3 complex–associated epigenetic coregulator rather than HDAC1/2 is responsible for SAP30/SIN3 coactivator function.

Gene transcriptional activation requires open chromatin and RNA polymerase II binding to the promoter. Next, we performed the assay for transposase-accessible chromatin using sequencing (ATAC-seq) and found that SAP30 KO decreased chromatin accessibility at SAP30/SIN3A/3B-coactivated genes in MDA-MB-231 cells (Figure 8F and Supplemental Figure 5, I and J). In contrast, the repressed genes *HSPA6* and *IPO13* became more accessible in SAP30-KO1 MDA-MB-231 cells (Supplemental Figure 5, G and H). Consistently, RNA polymerase II occupancy at SAP30/SIN3A/3B-coactivated genes was also significantly reduced by SAP30 KO in MDA-MB-231 cells (Figure 8G and Supplemental Figure 5, I and J). Collectively, these findings indicate that SAP30 regulates chromatin accessibility to increase RNA polymerase II occupancy at the promoters, leading to transcription of genes involved in angiogenesis and cell motility.

### SAP30 recruits MLL1 via its transactivation domain leading to transcriptional coactivation in breast cancer cells.

**Figure 9:**
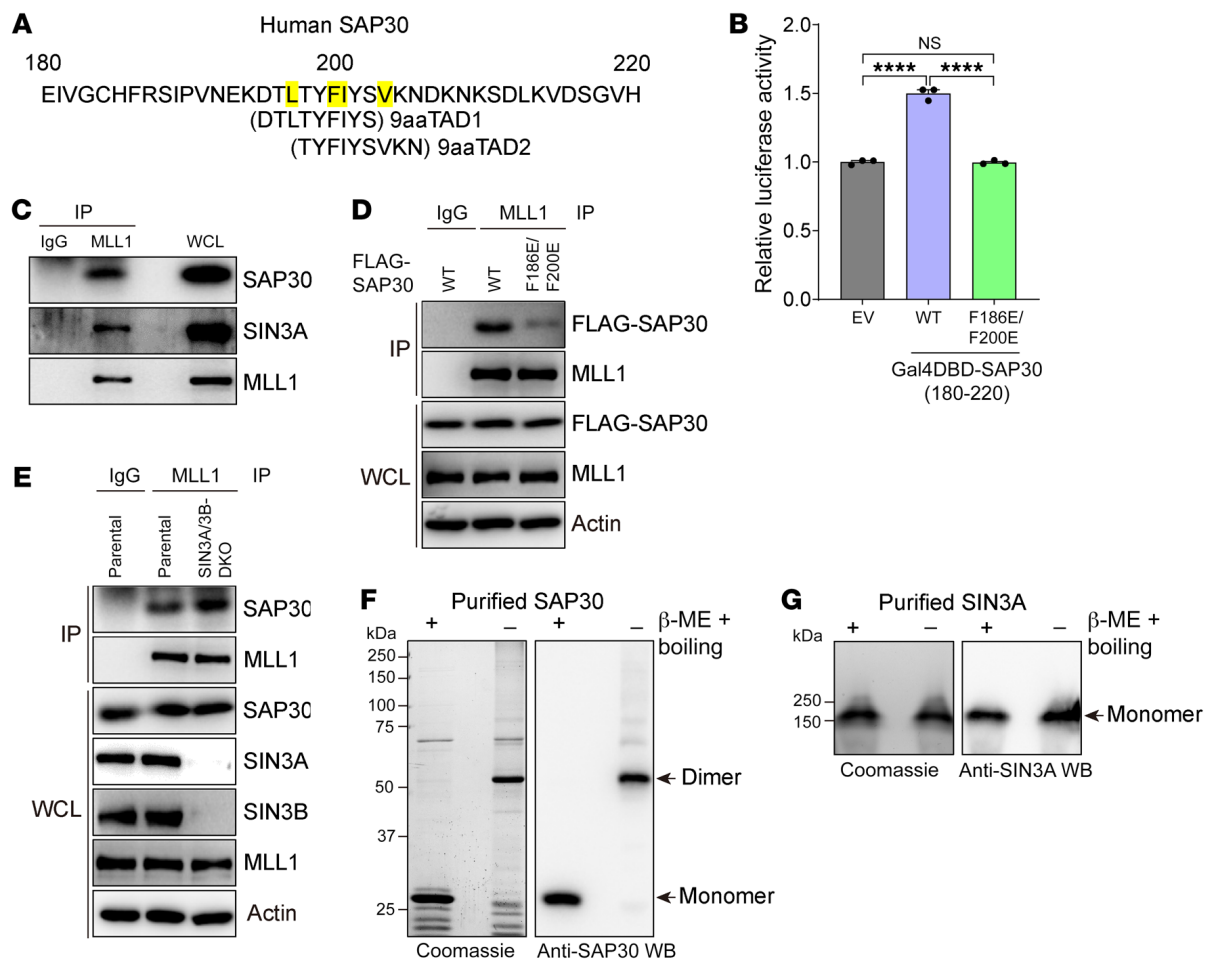
MLL1 binds the transactivation domain of SAP30. (**A**) Predicted SAP30 transactivation domain. The key hydrophobic residues for transactivation activity are highlighted in yellow. (**B**) Gal4 luciferase reporter assay in HEK293T cells transfected with indicated plasmids (mean ± SEM, *n* = 3). ****P < 0.0001 by 1-way ANOVA with Tukey’s test. (**C**) Co-IP assay showing that MLL1 interacts with SAP30 and SIN3A in MDA-MB-231 cells (*n* = 2). (**D**) Co-IP assay showing that MLL1 binds F186/F200 residues of SAP30 in MDA-MB-231 cells (*n* = 3). (**E**) Co-IP assay showing that SIN3A/3B DKO increases MLL1-SAP30 interaction in MDA-MB-231 cells (*n* = 2). SIN3A and SIN3B blots were run in parallel using the same biological samples. (**F** and **G**) Coomassie staining and immunoblot of purified human SAP30 (**F**, *n* = 3) and SIN3A (**G**, *n* = 2) protein treated with or without β-mercaptoethanol (β-ME) and boiling.

We analyzed the human SAP30 amino acid sequence with a 9aaTAD algorithm to search its potential transactivation domain (32). Two 9aaTAD motifs were identified within amino acids 195–206 of SAP30 (Figure 9A), identical to the conserved transactivation domains ϕ-×-×-ϕ-ϕ and ϕ-ϕ-×-×-ϕ (where ϕ represents hydrophobic residues and × represents any residues) found in p53, VP16, and EZH2 (33–35). The amino acid sequence of these potential SAP30 transactivation domains was highly conserved from yeast to mammals (Supplemental Figure 6A). To determine the transactivation activity of SAP30, we generated a 9aaTAD motif–containing 180–220 amino acids of SAP30 fused to Gal4 DNA-binding domain (DBD) and performed Gal4 luciferase reporter assay. Expression of Gal4DBD-SAP30 (180–220 aa) significantly increased the firefly/*Renilla* luciferase activity in HEK293T cells as compared with Gal4DBD alone (Figure 9B). F186E/F200E mutation abolished SAP30 transactivation activity in HEK293T cells, albeit its expression levels were much higher (Figure 9B and Supplemental Figure 6B). These findings provide direct evidence that SAP30 is a transcriptional coactivator.

To dissect the mechanism underlying SAP30-mediated transcriptional coactivation, we screened SAP30-interacting proteins with GST pull-down assay followed by mass spectrometry (MS). GST-tagged SAP30 was expressed and purified from bacteria and then incubated with nuclear lysates extracted from MDA-MB-231 cells. MS analysis identified that 226 proteins overlapped from 3 independent experiments were pulled down by GST-SAP30, suggesting that these proteins are potential SAP30-interacting proteins (Supplemental Figure 6C). Among these, we sorted out 13 epigenetic coregulators (Supplemental Figure 6D), of which KMT2A, also known as MLL1, was selected for further studies because 9aaTAD is known to interact with histone modifiers (32). To validate our screening results, we performed co-IP assay using anti-MLL1 antibody or IgG, showing that anti-MLL1 antibody but not IgG precipitated endogenous SAP30 and SIN3A besides MLL1 itself in MDA-MB-231 and SUM159 cells (Figure 9C and Supplemental Figure 6E). Interestingly, the SAP30-MLL1 interaction was inhibited by F186E/F200E mutation but increased by SIN3A/3B DKO in MDA-MB-231 cells (Figure 9, D and E), suggesting that MLL1 shares the binding residues of SAP30 with SIN3A/3B. However, MLL1 KO failed to influence SAP30 binding to SIN3A in MDA-MB-231 cells (Supplemental Figure 6F). We also found that SAP30 KO had no effect on MLL1 binding to SIN3A in MDA-MB-231 cells (Supplemental Figure 6G). We next assessed the oligomeric state of SAP30 and SIN3A to further biochemically dissect SIN3A-SAP30-MLL1 interaction. We detected dimeric SAP30 protein that was expressed and purified from bacteria under non-reducing conditions (Figure 9F). SAP30 homodimer was also supported by ab initio protein structural modeling (Supplemental Figure 6, H–K) (36). In contrast, SIN3A protein was monomeric under non-reducing conditions when it was expressed and purified from Sf9 cells (Figure 9G). Together, these results suggest that each subunit of homodimeric SAP30 respectively binds to MLL1 and SIN3A through F186/F200 residues in breast cancer cells.

**Figure 10:**
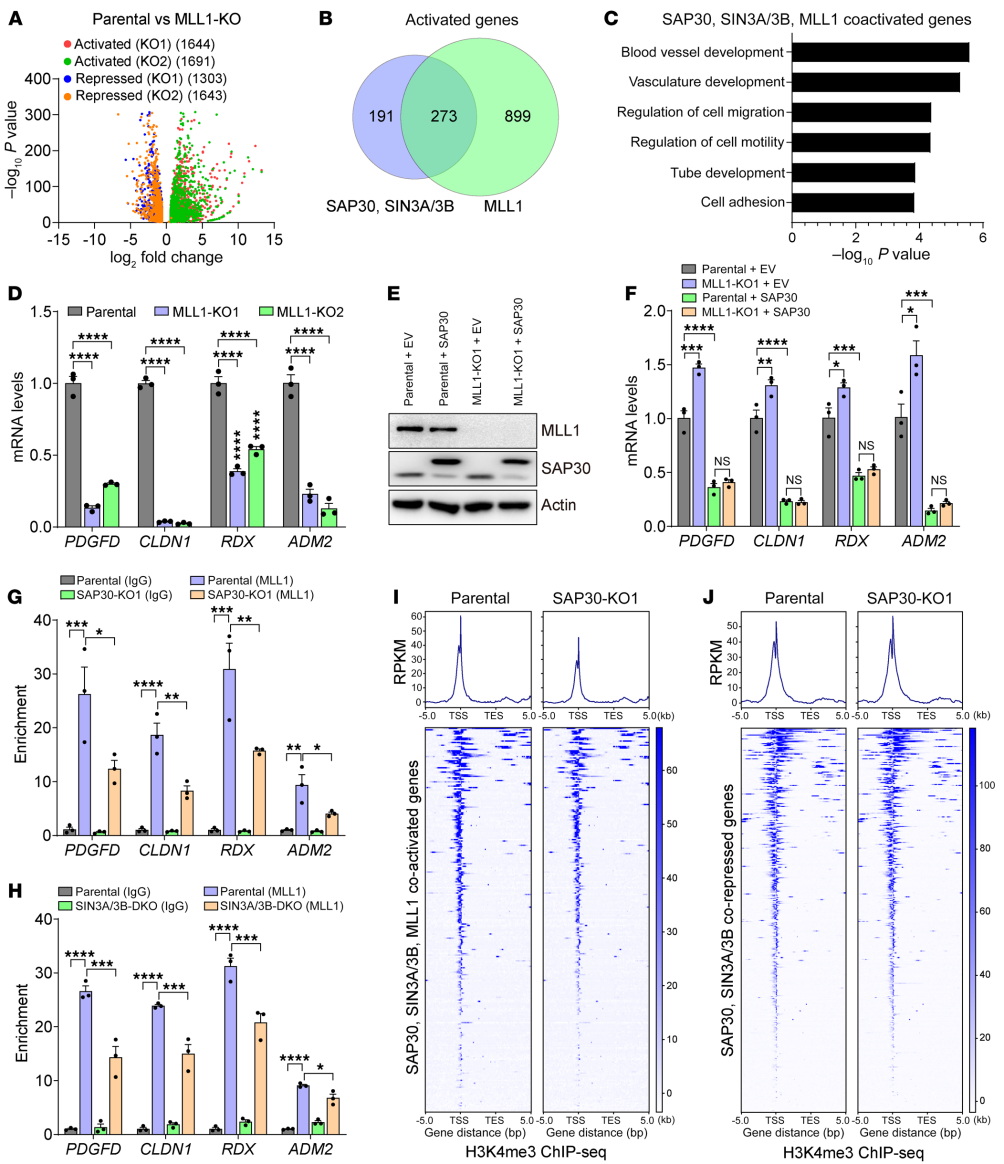
MLL1 is required for SAP30 coactivator function in breast cancer cells. (**A**) Volcano plot of MLL1 target genes in MDA-MB-231 cells (*n* = 2). (**B**) Venn diagram of overlapped activated genes by SAP30, SIN3A/3B, and MLL1 in MDA-MB-231 cells (*n* = 2). (**C**) Gene ontology analysis of SAP30-, SIN3A/3B-, and MLL1-coactivated genes in MDA-MB-231 cells (*n* = 2). (**D**) RT-qPCR analysis of indicated mRNAs in parental and MLL1-KO MDA-MB-231 cells (*n* = 3). (**E** and **F**) Immunoblot (**E**) and RT-qPCR (**F**) of indicated proteins or mRNAs in parental and MLL1-KO1 MDA-MB-231 cells overexpressing EV or SAP30 (*n* = 3). (**G** and **H**) ChIP-qPCR assay showing relative MLL1 occupancy on representative SAP30-, SIN3A/3B-coactivated genes in parental, SAP30-KO1 (**G**), and SIN3A/3B-DKO (**H**) MDA-MB-231 cells (*n* = 3). (**I** and **J**) Metaplot and heatmap of ChIP-Seq assay showing H3K4me3 occupancy on SAP30-, SIN3A/3B-, MLL1-coactivated genes (**I**) and SAP30-, SIN3A/3B-corepressed genes (**J**) in MDA-MB-231 cells (*n* = 2). Data are mean ± SEM. *P < 0.05, **P < 0.01, ***P < 0.001, ****P < 0.0001. One-way ANOVA with Dunnett’s test (**D**); 2-way ANOVA with Tukey’s test (**F**–**H**).

Next, we generated 2 independent MLL1-KO MDA-MB-231 cell lines with the CRISPR/Cas9 technique (Supplemental Figure 6L) and analyzed MLL1 transcriptome in these KOs and their parental cells with RNA-Seq. Volcano plots showed that an almost equal number of genes were either induced or repressed by MLL1 in MDA-MB-231 cells (Figure 10A). Importantly, a quarter of MLL1-induced genes overlapped with about 60% of SAP30/SIN3A/3B-coactivated genes (Figure 10B), and these overlapped genes were highly enriched in angiogenesis- and cell motility–related pathways (Figure 10C). RT-qPCR assay confirmed that SAP30-coactivated genes including *PDGFD*, *CLDN1*, *RDX*, and *ADM2* all were induced by MLL1 in MDA-MB-231 cells (Figure 10D). A similar pattern of gene regulation was also observed in SUM159 cells (Supplemental Figure 6, M and N). To determine whether MLL1 is required for SAP30 coactivator function, we generated parental and MLL1-KO1 MDA-MB-231 cells expressing SAP30 or empty vector (Figure 10E). RT-qPCR assay showed that ectopic expression of SAP30 significantly increased the transcription of *PDGFD*, *CLDN1*, *RDX*, and *ADM2*, which was abolished by MLL1 KO1 in MDA-MB-231 cells (Figure 10F). To determine whether SAP30/SIN3A/3B complex recruits MLL1 to coactivate gene transcription, we performed ChIP-qPCR using anti-MLL1 antibody or IgG and found that MLL1 was highly enriched at *PDGFD*, *CLDN1*, *RDX*, and *ADM2* in MDA-MB-231 cells, which was significantly inhibited by SAP30 KO1 or SIN3A/3B DKO (Figure 10, G and H). Although MLL1 bound the SAP30/SIN3A/3B-corepressed genes *ARTN*, *IPO13*, and *HSPA6*, SAP30 KO1 or SIN3A/3B DKO had no effect on MLL1 binding these genes in MDA-MB-231 cells (Supplemental Figure 6, O and P). These results indicate that the SAP30/SIN3A/3B complex specifically recruits MLL1 to its coactivated genes. Consistently, SAP30 KO1 reduced H3K4me3 enrichment at SAP30/SIN3A/3B/MLL1-coactivated genes but not SAP30/SIN3A/3B-corepressed genes in MDA-MB-231 cells (Figure 10, I and J, and Supplemental Figure 6, Q–T). Taken together, these results indicate that MLL1 interacts with SAP30/SIN3A and acts as a downstream executor to control the transcription of SAP30/SIN3-coactivated genes in breast cancer cells by increasing H3K4me3 levels.

### MLL1 is required for SAP30-mediated breast tumor growth and metastasis.

**Figure 11:**
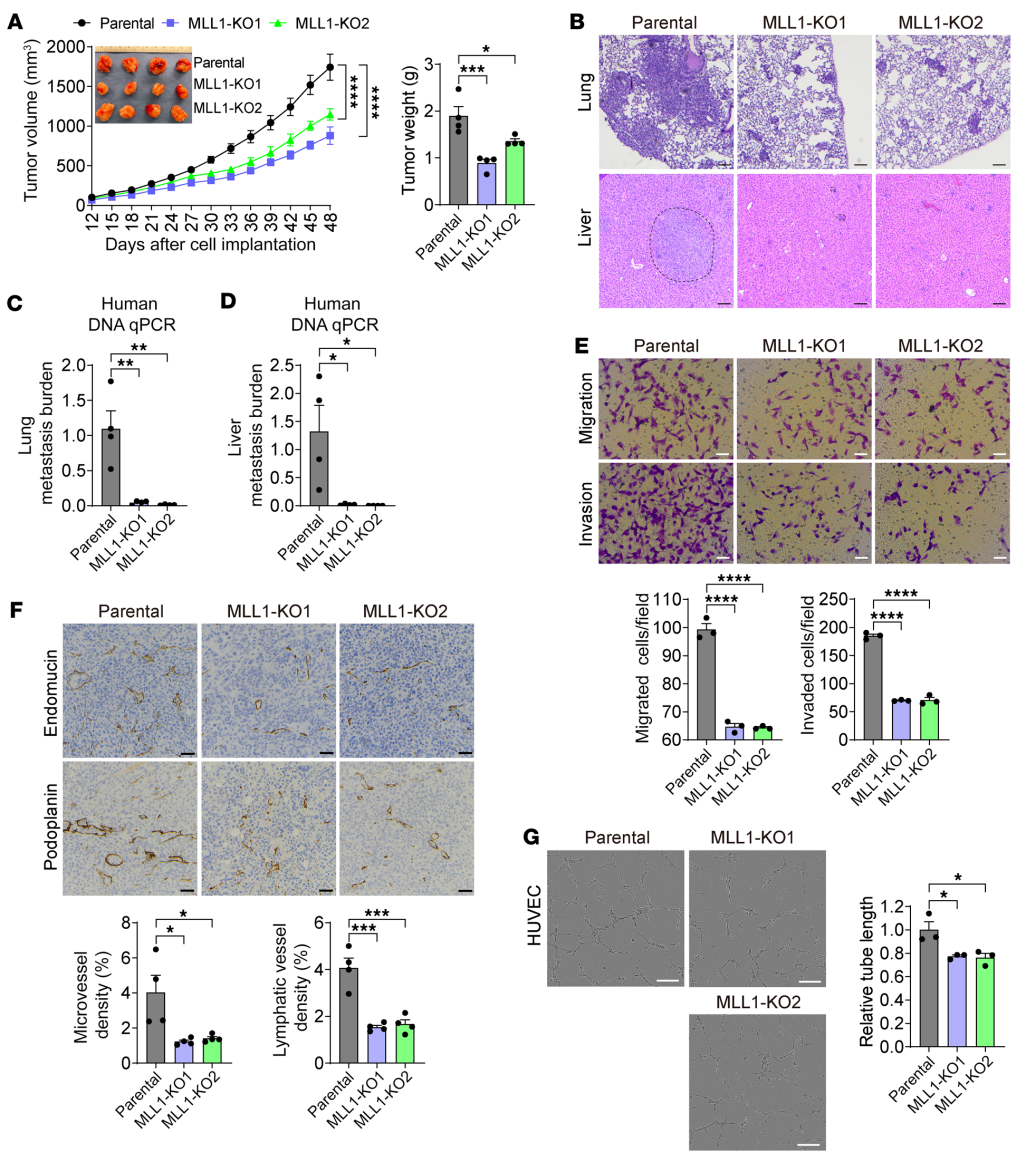
MLL1 promotes TNBC progression in mice. (**A**) Growth of parental and MLL1-KO1 or -KO2 MDA-MB-231 tumors in mice (*n* = 4). After harvesting, tumors were imaged (inset) and weighed (right). (**B**–**D**) Lung and liver metastasis in mice bearing parental and MLL1-KO1 or -KO2 MDA-MB-231 tumors by H&E staining (**B**) and qPCR assay (**C** and **D**). *n* = 4. (**E**) Migration and invasion of parental and MLL1-KO1 or -KO2 MDA-MB-231 cells (top) and their quantification (bottom). *n* = 3. (**F**) Immunohistochemical staining (top) and quantification (bottom) of endomucin and podoplanin in parental and MLL1-KO1 or -KO2 MDA-MB-231 tumors. *n* = 4. (**G**) In vitro angiogenesis of HUVECs incubated with conditional media from parental and MLL1-KO1 or -KO2 MDA-MB-231 cells (left). Total tube length is quantified (right). *n* = 3. Data are mean ± SEM. *P < 0.05, **P < 0.01, ***P < 0.001, ****P < 0.0001. One-way ANOVA with Dunnett’s test (**A**, right, and **C**–**G**); 2-way ANOVA with Dunnett’s (**A**, left). Scale bars: 100 μm (**B** and **G**), 50 μm (**E** and **F**).

Next, we investigated whether MLL1 promotes breast cancer progression. Both MLL1 KO1 and KO2 significantly attenuated MDA-MB-231 tumor growth and distant metastasis to the lungs and liver in the orthotopic xenograft mouse models (Figure 11, A–D). More robust inhibition of tumor growth and lung metastasis was observed in mice implanted with MLL1-KO1 or -KO2 SUM159 cells (Supplemental Figure 7, A–C). Boyden chamber assay showed that MLL1 KO1 or KO2 significantly inhibited migration and invasion of MDA-MB-231 and SUM159 cells in vitro (Figure 11E and Supplemental Figure 7D). We further found profound inhibition of angiogenesis and lymphangiogenesis by MLL1 KO1 or KO2 in vitro and in tumors (Figure 11, F and G). These results indicate that MLL1 phenocopies SAP30 to promote breast tumor angiogenesis, growth, and distant metastasis in vitro and in vivo.

**Figure 12:**
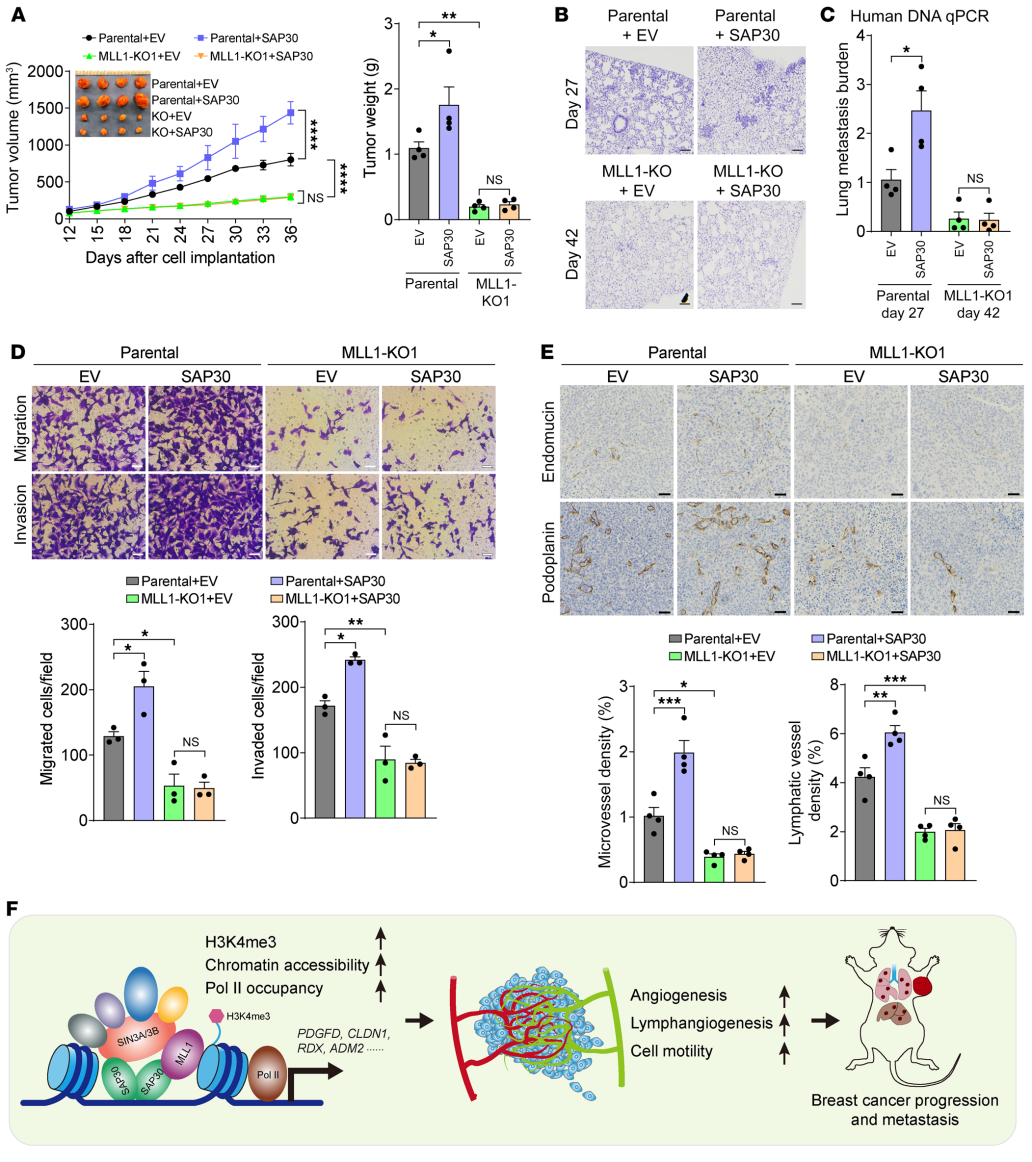
MLL1 is necessary for SAP30-mediated breast cancer progression. (**A**–**C**) Growth of EV- or SAP30-overexpressed parental and MLL1-KO1 MDA-MB-231 tumors and lung metastasis in mice (*n* = 4). After harvesting, tumors were imaged and weighed (**A**). Lung metastasis was assessed by H&E staining (**B**) and qPCR assay (**C**). (**D**) Migration and invasion of parental and MLL1-KO1 MDA-MB-231 cells overexpressing EV or SAP30 (top) and their quantification (bottom). *n* = 3. (**E**) Immunohistochemical staining (top) and quantification (bottom) of endomucin and podoplanin in EV- or SAP30-overexpressed parental and MLL1-KO1 MDA-MB-231 tumors. *n* = 4. (**F**) A proposed model of SAP30-mediated breast cancer progression, with SAP30 hijacking MLL1-dependent epigenetic reprogramming and gene activation. Data are mean ± SEM. *P < 0.05, **P < 0.01, ***P < 0.001, ****P < 0.0001. Two-tailed Student’s *t* test (**C**); 2-way ANOVA with Tukey’s test (**A**, **D**, and **E**). Scale bars: 100 μm (**B**), 50 μm (**D** and **E**).

Lastly, we investigated whether MLL1 is required for SAP30-mediated breast cancer progression. SAP30 overexpression significantly increased growth of parental MDA-MB-231 tumors but not MLL1 KO1 tumors in mice, even after an extended breeding of MLL1-KO1 tumor–bearing mice for an additional 2 weeks (Figure 12A and Supplemental Figure 7E). MLL1 KO1 also abolished SAP30-induced lung metastasis, tumor angiogenesis and lymphangiogenesis, and in vitro cell migration and invasion (Figure 12, B–E). Collectively, MLL1 loss-of-function effects on SAP30-mediated breast tumor growth and metastasis were supported by the in vitro and in vivo results from MLL1 binding–resistant SAP30 F186E/F200E mutant (Figure 5, A–H), and thus these combined results indicate that SAP30-mediated breast cancer progression is MLL1 dependent.

## Discussion

In the present study, for the first time to our knowledge, we identified an oncogenic activity of a previously unappreciated SAP30 protein in breast cancer. Rather than being a corepressor, SAP30 coordinates with SIN3A/3B and MLL1 to increase chromatin accessibility and coactivate the transcription of genes involved in cell motility, angiogenesis, and lymphangiogenesis, leading to breast tumor growth and metastasis to distant organs (Figure 12F).

Genomic analyses of human primary and metastatic breast tumors have identified genomic mutations and amplified gene foci that drive tumor growth and metastasis (3–7). Although the *SAP30* gene locus is not obviously amplified in human breast tumor, SAP30 is highly upregulated at the mRNA and protein levels in more than 50% of patients with all major molecular subtypes of breast cancer, particularly metastatic breast cancer, suggesting non-genetic regulation of SAP30 expression in breast cancer. Nonmutational epigenetic reprogramming is an emerging hallmark in human cancers (8). We and others have previously shown that epigenetic coregulators such as ZMYND8, CHD4, CARM1, TRIM24, and JMJD2C are frequently amplified or upregulated to promote breast cancer progression through their role in epigenetic reprogramming (37–42). Hypoxia plays an important role in upregulation of some of these epigenetic coregulators in breast cancer (37, 39). Although the molecular mechanism underlying SAP30 upregulation in breast tumors remains to be determined, our findings reveal that SAP30 is an independent prognostic factor for breast cancer and has a strong clinical relevance in patients with breast cancer.

Previous biochemical studies demonstrated that SAP30 is a core subunit of the SIN3 protein complex, although its function in the complex has not yet been fully characterized (10, 11). Our current studies reveal the dual activity of the SIN3 complex in regulation of gene silencing and activation in cancer cells; this activity supports previous studies in yeast and embryonic stem cells (29, 30, 43). A well-known mechanistic model of dual gene regulation has been proposed for type II nuclear receptors, which involves a switch from the corepressor to the coactivator binding to the nuclear receptor upon activation (44). Distinct to nuclear receptors, the canonical and the noncanonical SIN3 complex coordinates with its respective corepressors HDAC1/2 and coactivator MLL1 to control their unique sets of genes in breast cancer cells, and SAP30 plays a determinant role in this coordination. While SIN3A and SIN3B themselves do not have a DNA binding ability, we showed that SAP30 is required for recruitment of the SIN3 complex to the chromatin, regardless of corepressed and coactivated genes. Meanwhile, SAP30 controls recruitment of MLL1 selectively to coactivated genes. MLL1 catalyzes an active transcriptional mark H3K4me3, which is strongly associated with chromatin accessibility and gene transcription (45). Our findings suggest that, by hijacking MLL1, SAP30 increases chromatin accessibility and RNA polymerase II recruitment to turn on transcription of its coactivated genes in breast cancer cells.

Another fundamental discovery of this study is characterization of a putative transactivation domain at the C-terminus of SAP30, which directly supports a notion that SAP30 is a coactivator. As such, the SIN3 complex acquires the intrinsic coactivator function. Our biochemical studies revealed that SAP30 is a homodimeric protein with one subunit binding to SIN3A/3B and another subunit binding to MLL1. Intriguingly, both MLL1 and SIN3A bind the same Phe residues within the SAP30 transactivation domain. This binding model suggests that SAP30 acts as a molecular hub connecting with SIN3A and MLL1 to coordinate gene transcription in breast cancer cells (Figure 12F). Further protein structural studies are needed to confirm our hypothesis. Collectively, SAP30 is associated with 2 distinct types of histone modifiers, MLL1 and HDAC1/2, which alter the local chromatin state on their respective sets of genes to turn on or off their transcription, thereby evoking SIN3-dependent diverse biological responses. The regulatory mechanism underlying the SIN3 functional switch between gene coactivation and silencing awaits future investigation.

We showed here that SAP30 promotes cancer cell motility, angiogenesis, and lymphangiogenesis in mouse breast cancer models, which is consistent with its effect on gene induction. We further identified similar oncogenic phenotypes for MLL1 in breast cancer, providing functional evidence of the SAP30/SIN3/MLL1 complex as representative of a transcriptional dependency in breast cancer. Despite its effectiveness in tumor inhibition, loss of SAP30, SIN3, or MLL1 fails to eliminate primary tumors and distant metastasis in the mouse models. The extent to which these primary and metastatic breast tumors acquire the transcriptional dependency on the SAP30/SIN3/MLL1 complex remains uncertain. Cell migration and invasion, angiogenesis, and lymphangiogenesis are the important players in tumor growth and metastasis. Our present work uncovers an epigenetic mechanism underlying angiogenesis and cell motility in breast tumors. Although previous studies reported a controversial role of the SIN3 complex in tumor progression (14, 46–48), our studies clearly showed that the canonical corepressor function of the SIN3 complex is dispensable for breast cancer progression in mice. While several HDAC inhibitors are currently in clinical trials with breast cancer, the SAP30/SIN3 complex may not become a biomarker for guidance of HDAC inhibitor therapy in patients with breast cancer. Instead, breast tumors with high levels of SAP30 are most likely vulnerable to MLL1 inhibitor. Targeting MLL1-SAP30 protein interaction may be another effective approach for the treatment of this subtype of breast tumors. The TCGA data set demonstrates SAP30 upregulation in multiple human cancers. Therefore, our findings implicate a broad translational impact of SAP30 in human cancers.

In conclusion, our work identifies SAP30 as a clinically relevant target in breast cancer. SAP30 hijacks a prominent epigenetic regulator, MLL1, to enhance cancer cell motility, angiogenesis, and lymphangiogenesis, leading to breast tumor progression (Figure 12F). As such, these findings unveil a fundamental mechanism of epigenetic vulnerability that can provide therapeutic opportunities for the treatment of breast cancer.

## Methods

### Plasmid constructs.

sgRNAs targeting *SAP30*, *SIN3A*, *SIN3B*, and *MLL1* (Supplemental Table 1) were designed by the online CRISPR design program CRISPick (https://portals.broadinstitute.org/gppx/crispick/public) and cloned into BsmBI-linearized lentiCRISPRv2 vector (Addgene 52961). DNA oligonucleotides of shRNA targeting human *SAP30* (Supplemental Table 1) were annealed and ligated into AgeI/EcoRI-linearized pLKO.1 vector (Addgene 8435). Full-length human *SAP30* cDNA was PCR amplified and cloned into p3×FLAG-CMV-7 (MilliporeSigma) or pGex-6P-1 (GE Healthcare) vector. sgRNA1-resistant *SAP30* cDNA was generated by mutation of the PAM sequence 5′-GGG-3′ to 5′-GGA-3′ using PCR mutagenesis assay and subcloned into lentiviral cFugw-3×FLAG vector. F186E/F200E mutant SAP30 plasmid was generated by PCR mutagenesis assay. For Gal4 luciferase reporter assay, the cDNA fragment encoding WT or F186E/F200E SAP30 (180–220 aa) was inserted downstream of Gal4 DNA-binding domain coding sequence in pGal4DBD plasmid. Full-length and truncated *SIN3A* cDNAs were PCR amplified and inserted into lentiviral pLVX-FLAG or pFastBac1-10×His vector. pFastBac1-10×His-SIN3A plasmid was transformed into competent DH10Bac *E. coli* cells (Thermo Fisher Scientific) to generate recombinant bacmid DNA. All plasmids were confirmed by DNA sequencing.

### Cell culture.

MDA-MB-231 (a gift from Rolf Brekken, UT Southwestern), HEK293T, SUM159 (gifts from Gregg L. Semenza, Johns Hopkins University School of Medicine, Baltimore, Maryland, USA), and HEK293FT (Thermo Fisher Scientific) cells were cultured in high-glucose DMEM or DMEM/Ham’s F-12 (MilliporeSigma) supplemented with heat-inactivated 10% FBS (MilliporeSigma) at 37°C in a 5% CO_2_/95% air incubator. Human HUVECs (a gift from Ondine Cleaver, UT Southwestern) were cultured in M200PRF medium with low-serum growth supplement (Thermo Fisher Scientific) at 37°C in a 5% CO_2_/95% air incubator. HUVECs within the first 3 passages were used for experiments. Sf9 cells (a gift from Xuewu Zhang, UT Southwestern) were cultured in Sf-900 III SFM medium (Gibco) at 27°C in a shaking incubator.

### Lentivirus production.

The lentivirus was generated by transfection of HEK293FT cells with transducing vector and packaging vectors psPAX2 (Addgene 12260) and pMD2.G (Addgene 12259). Forty-eight hours after transfection, the supernatant containing virus particles was collected, filtered, and transduced into cancer cells.

### Generation of KO, KD, and rescue cell lines.

SAP30, SIN3A/3B, and MLL1 KO cell lines were generated using the CRISPR/Cas9 technique. Briefly, cells were transiently transfected with sgRNA vector using PolyJet (SignaGen Laboratories) or infected with sgRNA lentivirus. Forty-eight hours after transfection/transduction, cells were treated with puromycin (1 μg/mL) for 3 days. Multiple single KO cells were selected, amplified, mixed, and verified by immunoblot assay. SAP30 and SIN3A rescue cells were generated by infection of their individual KO cells with lentivirus encoding WT or mutant SAP30 or SIN3A, respectively. Expression of SAP30 and SIN3A in their rescue cells was verified by immunostaining and immunoblot assays. Luciferase-expressing cells were generated by infection of SC or SAP30-KO cells with lentivirus encoding luciferase, followed by hygromycin selection (500 μg/mL) and verification via immunoblot assay. SAP30-KD cells were generated by infection of cells with lentivirus encoding SAP30 shRNA, followed by puromycin selection (1 μg/mL) and verification via immunoblot assay.

### Cell proliferation assay.

MDA-MB-231 and SUM159 cells (2 × 10^5^ cells per well) were seeded onto a 6-well plate and cultured for 24, 48, and 72 hours. The cell number at each time point was determined by trypan blue assay.

### Immunoprecipitation and immunoblot assays.

Cells were lysed in NETN lysis buffer (150 mM NaCl, 1 mM EDTA, 10 mM Tris-HCl pH 8.0, 0.5% NP-40, and protease inhibitor cocktail) for 30 minutes on ice, followed by sonication. After centrifugation at 13,000*g* for 15 minutes, supernatant was collected for immunoprecipitation (IP) overnight with the following antibodies: SAP30 (Bethyl Laboratories, catalog A303-551A), MLL1 (Bethyl Laboratories, catalog A300-374A), or FLAG (MilliporeSigma, catalog F3165), in the presence of protein A/G magnetic beads (Bio-Rad). The next day, proteins bound on the beads were washed 4 times with NETN lysis buffer, boiled in 1× Laemmli buffer, and fractionated by SDS-PAGE, followed by immunoblot assay with the antibodies listed in Supplemental Table 2.

### Boyden chamber migration and invasion assays.

Cells (3 × 10^4^) were resuspended in serum-free medium and seeded in a Transwell insert (for migration) or a Matrigel-coated Transwell insert (for invasion) in the presence of cell culture medium with 10% FBS at the bottom chamber. Sixteen hours (migration) or 24 hours (invasion) later, cells that invaded to the lower side of the Transwell insert were fixed with methanol, stained with 0.1% crystal violet, and counted.

### In vitro angiogenesis assay.

A total of 4 × 10^4^ HUVECs were starved in M200PRF medium without low-serum growth supplement for 2 hours, resuspended in fresh conditional medium with 1% FBS, and seeded onto a 96-well plate coated with growth factor–reduced Matrigel (BD Biosciences). Conditional media were collected by incubation of MDA-MB-231 or SUM159 cells with serum-free DMEM for 24 hours. HUVECs were imaged using the IncuCyte S3 live-cell analysis system (Sartorius) for 5 hours, and the total tube length from 5 randomly selected fields at ×10 magnification was measured by Wimasis image analysis.

### PDGFD ELISA assay.

A total of 1 × 10^5^ cells were seeded onto a well of a 24-well plate, and the medium was harvested after 72-hour culture. The PDGFD levels in the conditional medium were measured using a PDGFD ELISA Kit (Biomatik) according to the manufacturer’s instructions.

### Immunohistochemistry assay.

Immunohistochemistry assays were performed by the Dako Autostainer Link 48 system. Briefly, the slides were baked, deparaffinized, and hydrated, followed by antigen retrieval in a Dako PT Link. The tissues were incubated with a peroxidase block and then one of the following primary antibodies: SAP30 (1:200; Bethyl Laboratories, catalog A303-551A), Ki67 (1:1,000; Proteintech, catalog 27309-1-AP), cleaved caspase-3 (1:1,500; Cell Signaling Technology, catalog 9661), endomucin (1:50; Santa Cruz Biotechnology, catalog sc-65495), or podoplanin (1:1,000; Abcam, catalog ab11936). The staining was visualized using the EnVision FLEX visualization system (Agilent). The H-scores of SAP30 staining were calculated with Qupath software.

### Luciferase reporter assay.

HEK293T cells were seeded onto a 48-well plate and transfected with empty vector pGal4DBD, Gal4DBD-WT SAP30 (180–220 aa), or Gal4DBD-F186E/F200E SAP30 (180–220 aa), pG5E1bLuc reporter plasmid, and control pSV-Renilla reporter plasmid. The firefly and *Renilla* luciferase activities were measured 48 hours after transfection by the Dual-Luciferase Assay System (Promega).

### RT-qPCR assay.

Total RNA was isolated from the cultured cells using TRIzol (Thermo Fisher Scientific), treated with DNase I (Thermo Fisher Scientific), and then subjected to cDNA synthesis with the iScript cDNA Synthesis Kit (Bio-Rad). qPCR was performed with the primers (Supplemental Table 3) and iTaq Universal SYBR Green Supermix (Bio-Rad) and normalized to the internal control 18S RNA as described previously (49).

### RNA-Seq assay.

Total RNA was isolated from cultured parental, SAP30-KO1/KO2, SIN3A/3B-DKO1/DKO2, and MLL1-KO1/KO2 MDA-MB-231 cells using the RNeasy Mini Kit and treated with DNase (Qiagen). The quality of total RNA was confirmed with an RNA integrity number score 8.5 or higher by the Agilent Tapestation 4200. RNA-Seq libraries were prepared with KAPA mRNA Hyper Prep (Roche) and sequenced with an Illumina NextSeq 500. Bioinformatics analysis was performed as described previously (37, 38).

### ChIP-qPCR assay.

Parental, SAP30-KO1, or SIN3A/3B-DKO MDA-MB-231 cells were cross-linked with 1% formaldehyde for 20 minutes at room temperature and quenched in 0.125 M glycine. Chromatin was isolated and digested using the SimpleChIP Enzymatic Chromatin IP Kit (Cell Signaling Technology) and subjected to IP overnight with antibodies against SAP30 (Bethyl Laboratories, catalog A303-551A), SIN3A (Abcam, catalog ab3479), SIN3B (Novus Biologicals, catalog NBP2-20367), MLL1 (Bethyl Laboratories, catalog A300-374A), HDAC2 (Proteintech, catalog 12922-3-AP), or IgG at 4°C. Precipitated chromatin DNA was extensively washed, eluted, and subjected to reverse cross-linking and proteinase K treatment at 65°C for 4 hours. ChIP DNA was column-purified and quantified by real-time qPCR assay with the specific primers (Supplemental Table 4). Fold enrichment was calculated based on Ct as 2^–Δ(ΔCt)^, where ΔCt = Ct_IP_ – Ct_input_ and Δ(ΔCt) = ΔCt_antibody_ – ΔCt_IgG_.

### ChIP-Seq assay.

Parental and SAP30-KO1 MDA-MB-231 cells were cross-linked with 1% formaldehyde for 20 minutes at room temperature and quenched in 0.125 M glycine. Cells were lysed in cell lysis buffer (10 mM Tris-HCl pH8.0, 10 mM EDTA, 100 mM NaCl, 0.25% Triton X-100, protease inhibitor cocktail). The nuclei were lysed in nuclear lysis buffer (50 mM HEPES-KOH pH 7.5, 1 mM EDTA, 150 mM NaCl, 1% Triton X-100, 0.1% sodium deoxycholate, 1% SDS, protease inhibitor cocktail), and chromatin was pelleted by centrifugation. The chromatin was then sonicated and subjected to IP overnight in the presence of Protein G Dynabeads (Thermo Fisher Scientific) with antibodies against SAP30 (Bethyl Laboratories, catalog A303-551A), SIN3A (Abcam, catalog ab3479), SIN3B (Novus Biologicals, catalog NBP2-20367), RNA polymerase II (Abcam, catalog ab817), H3K4me3 (Cell Signaling Technology, catalog 9751), H3K9ac (Abcam, catalog ab10812), or histone H3 (Cell Signaling Technology, catalog 4620) at 4°C. Precipitated chromatin DNA was extensively washed and eluted with the freshly prepared elution buffer (0.1 M NaHCO_3_, 1% SDS). Reverse cross-linking was performed at 67°C for 6 hours followed by treatment with proteinase K. ChIP DNA was purified with phenol/chloroform/isoamyl alcohol (25:24:1, vol/vol) followed by treatment with RNase A and used for library preparation with a ThruPLEX DNA-Seq kit (Takara). ChIP-Seq libraries were sequenced with the Illumina NextSeq 500. Bioinformatics analysis was performed as described previously (37, 38).

### ATAC-seq assay.

ATAC-seq was performed as described previously (50). Briefly, a total of 50,000 parental and SAP30-KO1 MDA-MB-231 cells were washed once with 50 μL cold PBS and resuspended in 50 μL lysis buffer (10 mM Tris-HCl pH 7.4, 10 mM NaCl, 3 mM MgCl_2_, 0.1% IGEPAL CA-630). The suspension was then centrifuged at 500*g* for 5 minutes at 4°C, followed by addition of 50 μL transposition reaction mix of Nextera XT Index Kit (FC-121-1001, Illumina). After incubation for 30 minutes at 37°C, DNA was purified using a MinElute PCR Purification Kit (Qiagen). The transposed DNA fragments were then subjected to 5 cycles of PCR for preamplification and further amplified by PCR for 5 cycles. The amplified libraries at the size of 200–500 bp were purified by gel size selection with a MinElute Gel Extraction Kit (Qiagen), quantified with a Qubit 2.0 Fluorometer (Thermo Fisher Scientific), and sequenced on the Illumina NextSeq 500 with the read configuration as 75 bp, single end. Fastq files were subjected to quality check using fastqc (v0.11.2) and fastq_screen (v0.4.4). Low-quality reads and adapter were removed using Trim Galore (v0.4.5). The remaining reads were aligned to the human genome (hg19) using bowtie2 (v2.3.3.1). MarkDuplicates module in Picard (v2.10.10) was used to remove duplicate alignments. Peak calling was performed using MACS2 (v2.1.0) with a *q* value threshold of 0.05. The metagene analysis was performed using the computeMatrix and plotHeatmap modules in deepTools (v3.1.1).

### Protein expression and purification.

GST-SAP30 fusion protein was expressed in *E. coli* BL21-Gold (DE3) and purified with glutathione-Sepharose beads (GE Healthcare) as described previously (49). The protein-bound beads were either immediately used for GST pull-down assay or subjected to GST removal with PreScission Protease (Cytiva) for protein oligomerization analysis in SDS-PAGE gel. Baculovirus carrying His-tagged SIN3A was produced by transfection of Sf9 cells with the bacmid DNA using Lipofectamine 3000 (Thermo Fisher Scientific). Recombinant His-SIN3A protein was expressed in Sf9 cells and extracted using lysis buffer (20 mM sodium phosphate, 300 mM NaCl, 10 mM imidazole, pH 7.4) with sonication. After centrifugation, the supernatant was incubated by rotation with Ni-NTA magnetic beads (New England Biolabs) for 1 hour at 4°C. The protein-bound beads were then washed with washing buffer (20 mM sodium phosphate, 300 mM NaCl, 20 mM imidazole, pH 7.4) and eluted with elution buffer (20 mM sodium phosphate, 300 mM NaCl, 500 mM imidazole, pH 7.4). His-SIN3A protein was separated in SDS-agarose (1%) gel for protein oligomerization analysis.

### GST pull-down and MS assays.

GST-SAP30 fusion protein immobilized on glutathione-Sepharose beads was incubated with nuclear lysates of MDA-MB-231 cells for 6 hours at 4°C. The bound proteins were fractionated by SDS-PAGE, stained with Coomassie blue, excised, reduced with DTT, alkylated with iodoacetamide, and digested overnight with trypsin (Pierce). The tryptic peptides then underwent solid-phase extraction cleanup with an Oasis HLB plate (Waters) and were analyzed with an Orbitrap Fusion Lumos mass spectrometer coupled to an Ultimate 3000 RSLC-Nano liquid chromatography system. Samples were injected onto a 75 μm–inside diameter, 75 cm–long EasySpray column (Thermo Fisher Scientific) with buffer A (2% [vol/vol] acetonitrile and 0.1% formic acid in water) and eluted with a gradient from 0% to 28% of buffer B (80% [vol/vol] acetonitrile, 10% [vol/vol] trifluoroethanol, and 0.1% formic acid in water) for more than 90 minutes. The mass spectrometer operated in positive ion mode with a source voltage of 1.5 kV and an ion transfer tube temperature of 275°C. MS scans were acquired at a resolution of 120,000 in the Orbitrap, and up to 10 MS/MS spectra were obtained in the ion trap for each full spectrum acquired using higher-energy collisional dissociation for ions with charges 2 to 7. Dynamic exclusion was set for 25 seconds after an ion was selected for fragmentation. Raw MS data were analyzed using Proteome Discoverer v2.2 (Thermo Fisher Scientific), with peptide identification performed using Sequest HT searching against the human protein database from UniProt. Fragment and precursor tolerances of 10 ppm and 0.6 Da were specified, and 3 missed cleavages were allowed. Carbamidomethylation of cysteine was set as a fixed modification, with oxidation of methionine set as a variable modification. The false discovery rate cutoff was 1% for all peptides.

### Animal studies.

*Sap30^–/–^* mice were generated by the transgenic core at UT Southwestern Medical Center. Fertilized C57BL/6N oocytes were microinjected with cocktail containing Cas9 protein and 2 individual *Sap30* gRNAs (annealed crRNA and tracRNA; Supplemental Table 5) and implanted into pseudopregnant female Institute of Cancer Research (ICR) mice. F_0_ founders were crossed with C57BL/6J (The Jackson Laboratory, stock 000664) to generate *Sap30^–/–^* mice. *Sap30^–/–^* mice were genotyped and confirmed by Sanger sequencing. To study the roles of SAP30 in mammary tumor initiation and progression, *Sap30^–/–^* mice were crossed with MMTV-PyMT mice [B6.FVB-Tg(MMTV-PyVT)634Mul/LellJ; The Jackson Laboratory, stock 022974]. Tumor initiation time was determined with palpation and measurement (diameter ≥2 mm). All tumors were harvested, counted, and weighed at postnatal day 155. Lungs were perfused with PBS, inflated with 0.5% agarose, fixed in formalin, embedded in paraffin, and analyzed by H&E staining.

For the orthotopic breast cancer mouse model, 2 × 10^6^ cells in 100 μL PBS/Matrigel (1:1; Corning) were injected into the second left mammary fat pad of 6- to 8-week-old female NSG or NOD/SCID mice (The Jackson Laboratory). Tumor volume was measured with a caliper every 3 days beginning on day 12 to 15 after cell implantation and calculated according to the formula: volume = 0.52 × length × height × width. After mice were perfused with PBS and inflated with 0.5% agarose in the lungs, lungs and liver were harvested and analyzed by H&E staining and qPCR assays with primers specific for the human *HK2* gene and mouse and human 18S rRNA. Genomic DNA was extracted from peripheral blood in NSG mice bearing parental or SAP30-KO MDA-MB-231 tumors with a QIAamp DNA Blood Mini Kit (Qiagen) and used for quantification of CTCs by qPCR assay with primers specific for the human *HK2* gene and mouse and human 18S rRNA. MDA-MB-231 cells were mixed with blood from tumor-free NSG mice to generate a standard curve. The number of CTCs in mouse blood was calculated according to the standard curve.

For the tail vein injection model, 1 × 10^6^ cells in 100 μL PBS were injected into the tail vein of female NSG or NOD/SCID mice. Three weeks later, the lungs were perfused with PBS and subjected to qPCR assay with primers specific for the human *HK2* gene and mouse and human 18S rRNA.

Bioluminescence was imaged weekly by the SPECTRAL AMI-HTX imaging system after 15 mg/mL luciferin (GoldBio) was administered to mice intraperitoneally. The lungs were harvested at the end of the experiments, followed by ex vivo bioluminescence imaging with 300 μg/mL luciferin solution in a 24-well plate.

### Human breast tumor studies.

Human ER^+^, HER2^+^ breast tumors and their adjacent normal breast tissues, paired primary/metastatic breast tumors, and TNBC tissue microarray (TMA) were obtained from a surgical breast cancer pathologist and the UT Southwestern tissue management core. After SAP30 immunohistochemistry assay, H-scores were calculated with QuPath. For TNBC TMA, each staining was scored using 4 grades (none as 0, weak as 1, moderate as 2, and strong as 3) according to the percentage of SAP30-positive cells and immunostaining intensity.

### Statistics.

Statistical analysis was performed by 2-tailed Student’s *t* test between 2 groups, and 1-way or 2-way ANOVA with multiple testing correction within multiple groups. Quantification of SAP30 protein levels between normal breast tissues and human TNBC tissues was determined by χ^2^ test. Kaplan-Meier survival curve and tumor initiation were analyzed by log-rank test. The number of biological samples/experiments is shown in figures or figure legends. Data are expressed as mean ± SEM. *P* less than 0.05 was considered significant.

### Study approval.

Animal experiments were approved by the Animal Care and Use Committee at UT Southwestern Medical Center. The deidentified human ER^+^ breast tumors, HER2^+^ breast tumors, TNBC, and adjacent normal breast tissues were used for immunohistochemistry assay, which was approved by the Institutional Review Board at UT Southwestern Medical Center with informed consent.

### Data availability.

The RNA-Seq, ChIP-Seq, and ATAC-seq data were deposited at the Gene Expression Omnibus with accession numbers GSE216345, GSE216344, and GSE216342. Values for all data points in graphs are reported in the Supporting Data Values file.

## Author contributions

WL and YW conceived the study, analyzed the data, and wrote the paper. LB performed most experiments, analyzed the data, and wrote the paper. AK and CX performed bioinformatics analysis. MZ generated plasmids. YP provided tumor tissues and analyzed TMA. JEW performed mouse breeding and lentivirus production. All authors read and approved the manuscript.

## Supplementary Material

Supplemental data

Supporting data values

## Version 1 09/01/2023

Electronic publication
