# Supplementary material for: SAP30 promotes breast tumor progression by bridging the transcriptional corepressor SIN3 complex and MLL1
Source: J Clin Invest. 2023 Sep 1;133(17):e168362. doi: 10.1172/JCI168362 (PMC10471174; doi:10.1172/JCI168362)
Supplement: Supplemental data [file jci-133-168362-s152.pdf]

SAP30 promotes breast tumor progression by bridging the transcriptional corepressor SIN3 complex and MLL1

Lei Bao[1], Ashwani Kumar[2], Ming Zhu[1], Yan Peng[1], Chao Xing[2,3], Jennifer E. Wang[1], Yingfei Wang[1,4,5,6], Weibo Luo[1,7*]

[1]Department of Pathology,

[2]Eugene McDermott Center for Human Growth and Development,

[3]Department of Bioinformatics,

[4]Department of Neurology,

[5]Peter O'Donnell Jr. Brain Institute,

[6]Cecil H. and Ida Green Center for Reproductive Biology Sciences,

[7]Department of Pharmacology,

UT Southwestern Medical Center, Dallas, TX 75390, USA.

*****Address correspondence to: Weibo Luo, Department of Pathology, UT Southwestern Medical Center. 5323 Harry Hines Blvd., NB6.460, Dallas, TX 75390-9072, USA. Phone: 214.645.4770; E-mail: Weibo.Luo@UTSouthwestern.edu.

Short title: SAP30/SIN3/MLL1 complex promotes breast tumor progression

Supplemental Table 1. Oligonucleotide sequence used for sgRNAs and shRNA

SAP30 sgRNA1	5'-CTGACGAGATGAGCCGCGGC-3'
SAP30 sgRNA2	5'-ACGCCTGACGAGATGAGCCG-3'
SIN3A sgRNA	5'-GAAGCGGCGTTTGGATGACC-3'
SIN3B sgRNA	5'-AGAAGACGCCCTCACCTATC-3'
MLL1 sgRNA1	5'-AGGGGTCTTAATGATCCGCG-3'
MLL1 sgRNA2	5'-CTGTGGAGGTATCAACACTG-3'
SAP30 shRNA	5'-GCACAACTTGTTGAGATAGTT-3'

Supplemental Table 2. The list of antibodies used in this paper

Antibodies	Source	Identifier
anti-SAP30	Bethyl Laboratories	Cat# A303-551A; RRID: AB_10951854
anti-Endomucin	Santa Cruz Biotechnology	Cat# SC-65495; RRID: AB_2100037
anti-Podoplanin	Abcam	Cat# ab11936; RRID: AB_298718
anti-Actin	Proteintech	Cat# 66009-1-Ig; RRID: AB_2687938
anti-FLAG	Sigma-Aldrich	Cat# F3165; RRID: AB_259529
anti-SIN3A	Abcam	Cat# ab3479; RRID: AB_303839
anti-SIN3B	Novus Biologicals	Cat# NBP2-20367; RRID: N/A
anti-HDAC1	Bethyl Laboratories	Cat# A300-713A; RRID: AB_533395
anti-HDAC2	Proteintech	Cat# 12922-3-AP; RRID: AB_2118516
anti-MLL1	Bethyl Laboratories	Cat# A300-374A; RRID: AB_345243
anti-RNA polymerase II CTD repeat YSPTSPS	Abcam	Cat# ab817; RRID: AB_306327
anti-H3K9ac	Abcam	Cat# ab10812; RRID: AB_297491
anti-H3K4me3	Cell Signaling Technology	Cat# 9751; RRID: AB_2616028
anti-Histone H3	Cell Signaling Technology	Cat# 4620; RRID: AB_1904005
anti-Ki67	Proteintech	Cat# 27309-1-AP; RRID: AB_2756525
anti-cleaved caspase-3	Cell Signaling Technology	Cat# 9661; RRID: AB_2341188
Normal mouse IgG antibody	Santa Cruz Biotechnology	Cat# SC-2025; RRID: AB_737182
Normal rabbit IgG antibody	Cell Signaling Technology	Cat# 2729; RRID: AB_1031062

Supplemental Table 3. Oligonucleotide sequence used for qPCR primers

Gene	Forward primer sequence	Reverse primer sequence
HK2	5'-CCAGTTCATTCACATCATCAG-3'	5'-CTTACACGAGGTCACATAGC-3'
18S rRNA	5'-CGGCGACGACCCATTCGAAC-3'	5'-GAATCGAACCCTGATTCCCCGTC-3'
PDGFB	5'-CTCGATCCGCTCCTTTGATGA-3'	5'-CGTTGGTGCGGTCTATGAG-3'
PDGFD	5'-TTGTACCGAAGAGATGAGACCA-3'	5'-GCTGTATCCGTGTATTCTCCTGA-3'
CLDN1	5'-CCTCCTGGGAGTGATAGCAAT-3'	5'-GGCAACTAAAATAGCCAGACCT-3'
RDX	5'-TATGCTGTCCAAGCCAAGTATG-3'	5'-CGCTGGGGTAGGAGTCTATCA-3'
ADM2	5'-CTGAGCCCCATCTGAAGCC-3'	5'-CAGCACTGCGTGTAGACCAG-3'
NOTCH3	5'-CGTGGCTTCTTTCTACTGTGC-3'	5'-CGTTCACCGGATTTGTGTCAC-3'

Supplemental Table 4. Oligonucleotide sequence used for ChIP-qPCR primers

Gene	Forward primer sequence	Reverse primer sequence
PDGFD	5'-TCCTGCATGCTGAACTTTCC-3'	5'-TTGTTTGTCCCGTCACCATTTA-3'
CLDN1	5'-CGCCGGCATAGGAGTAAAT-3'	5'-AGCTGTTGGGCTTCATTCT-3'
RDX	5'-CCAAATGAAGCGTGAGACTTG-3'	5'-ATCTAAATTCCGCTCAGGTAGG-3'
ADM2	5'-TTGCATCAGCCTCCTCT-3'	5'-TGGACTCACCTGGGTTT-3'
ARTN	5'-CACTTGCCTGTTTCTGGT-3'	5'-AGAGGGAGGATGCAAGAA-3'
IPO13	5'-TCAACTCCACGGACTCTT-3'	5'-TCTCGGCTGCTATGAACA-3'
HSPA6	5'-GCTGGCTGCAGAGAAAC-3'	5'-CTGAAGCTTCTTGTCGGATG-3'
CXCR4	5'-CTCTTGCCATCCTCGTGTTTA-3'	5'-CGGGAGAGTGAGGAAATGAAA-3'

Supplemental Table 5. Oligonucleotide sequence used for generation and identification of *Sap30*$^{-/-}$ mice.

Left crRNA	/AltR1/rUrUrArGrUrArArArArUrGrCrArUrArGrGrCrC rCrGrUrUrUrUrArGrArGrCrUrArUrGrCrU/AltR2/
Right crRNA	/AltR1/rGrArArArCrArUrCrUrArUrCrUrGrCrCrArCrCr UrGrUrUrUrUrArGrArGrCrUrArUrGrCrU/AltR2/
Sap30-KO genotyping primer forward	5'-TCATTCGGCCTTTAATTGTAGCC-3'
Sap30-KO genotyping primer reverse	5'-GAACTGAAGTCCCATACCAAGC-3'
PyMT genotyping primer forward	5'-GGAAGCAAGTACTTCACAAGGG-3'
PyMT genotyping primer reverse	5'-GGAAAGTCACTAGGAGCAGGG-3'
PyMT internal control primer forward	5'-CAAATGTTGCTTGTCTGGTG-3'
PyMT internal control primer reverse	5'-GTCAGTCGAGTGCACAGTTT-3'

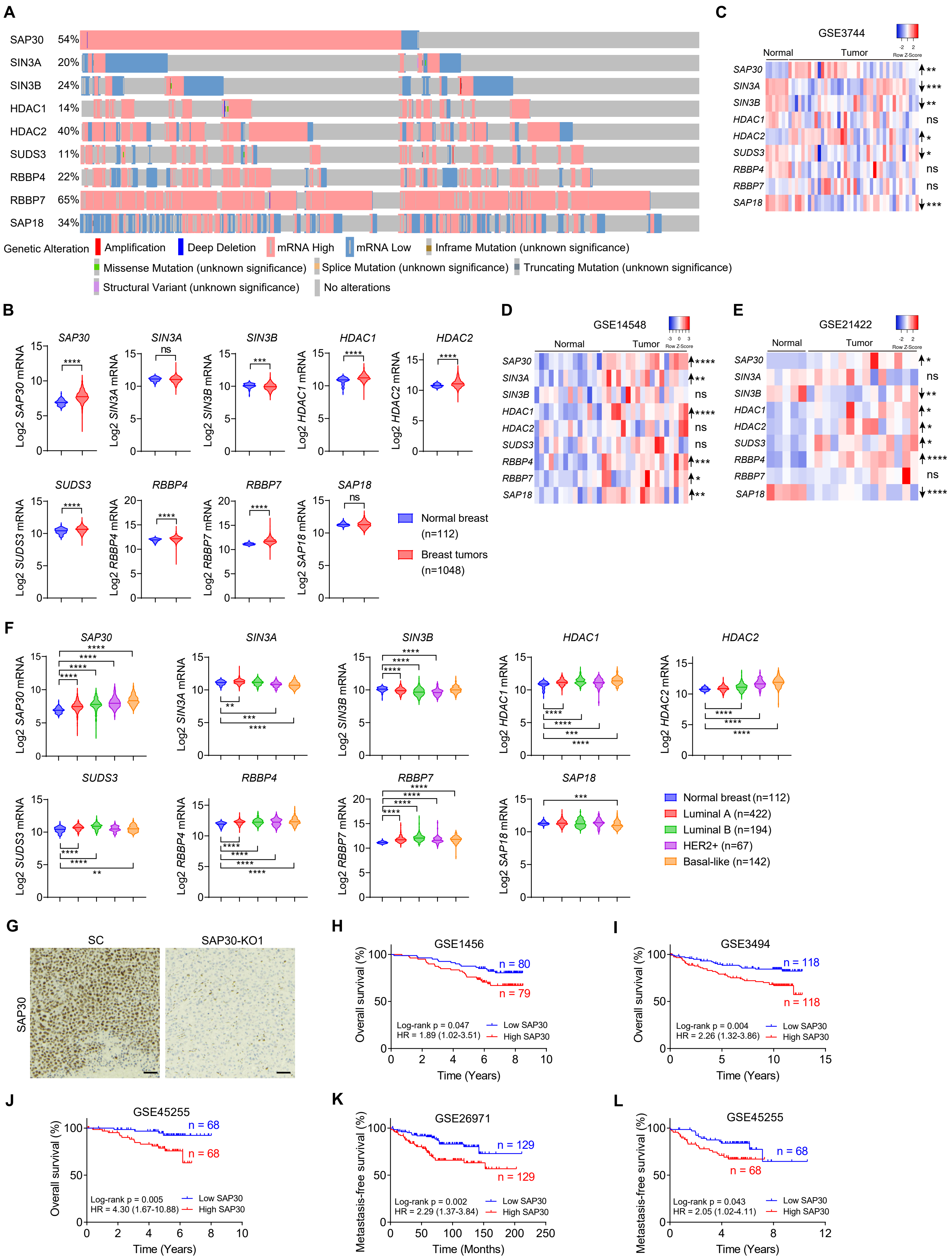

A
SAP30 54%
SIN3A 20%
SIN3B 24%
HDAC1 14%
HDAC2 40%
SUDS3 11%
RBBP4 22%
RBBP7 65%
SAP18 34%
Genetic Alteration
Amplification
Deep Deletion
mRNA High
mRNA Low
Inframe Mutation (unknown significance)
Missense Mutation (unknown significance)
Splice Mutation (unknown significance)
Truncating Mutation (unknown significance)
Structural Variant (unknown significance)
No alterations
C
GSE3744
Normal
Tumor
Row Z-Score
SAP30 **
SIN3A ***
SIN3B **
HDAC1 ns
HDAC2 *
SUDS3 *
RBBP4 ns
RBBP7 ns
SAP18 ***
B
SAP30
SIN3A
SIN3B
HDAC1
HDAC2
SUDS3
RBBP4
RBBP7
SAP18
Log2 SAP30 mRNA
Log2 SIN3A mRNA
Log2 SIN3B mRNA
Log2 HDAC1 mRNA
Log2 HDAC2 mRNA
Log2 SUDS3 mRNA
Log2 RBBP4 mRNA
Log2 RBBP7 mRNA
Log2 SAP18 mRNA
Normal breast (n=112)
Breast tumors (n=1048)
D
GSE14548
Normal
Tumor
Row Z-Score
SAP30 ****
SIN3A **
SIN3B ns
HDAC1 ****
HDAC2 ns
SUDS3 ns
RBBP4 ***
RBBP7 *
SAP18 **
E
GSE21422
Normal
Tumor
Row Z-Score
SAP30 *
SIN3A ns
SIN3B **
HDAC1 *
HDAC2 *
SUDS3 *
RBBP4 ****
RBBP7 ns
SAP18 ****
F
SAP30
SIN3A
SIN3B
HDAC1
HDAC2
SUDS3
RBBP4
RBBP7
SAP18
Normal breast (n=112)
Luminal A (n=422)
Luminal B (n=194)
HER2+ (n=67)
Basal-like (n=142)
G
SC
SAP30-KO1
SAP30
H
GSE1456
n = 80
n = 79
Log-rank p = 0.047
HR = 1.89 (1.02-3.51)
Low SAP30
High SAP30
Overall survival (%)
Time (Years)
I
GSE3494
n = 118
n = 118
Log-rank p = 0.004
HR = 2.26 (1.32-3.86)
Low SAP30
High SAP30
Overall survival (%)
Time (Years)
J
GSE45255
n = 68
n = 68
Log-rank p = 0.005
HR = 4.30 (1.67-10.88)
Low SAP30
High SAP30
Overall survival (%)
Time (Years)
K
GSE26971
n = 129
n = 129
Log-rank p = 0.002
HR = 2.29 (1.37-3.84)
Low SAP30
High SAP30
Metastasis-free survival (%)
Time (Months)
L
GSE45255
n = 68
n = 68
Log-rank p = 0.043
HR = 2.05 (1.02-4.11)
Low SAP30
High SAP30
Metastasis-free survival (%)
Time (Years)

Figure S1. SAP30 is upregulated in human breast tumors and negatively correlated with survival of breast cancer patient.

(**A**) Genetic aberrations of the core subunits of SIN3 complex in human breast tumors. Data were retrieved from TCGA breast invasive carcinoma dataset (PanCancer Atlas) at cBioPortal ($n = 994$).

(**B**) mRNA expression analysis of the core subunits of SIN3 complex in human breast tumors and normal breast tissues from TCGA breast invasive carcinoma dataset. ***$P < 0.001$, ****$P < 0.0001$, by two-tailed Student's t test. ns, not significant.

(**C**-**E**) mRNA expression analysis of the core subunits of SIN3 complex in human breast tumors and normal breast tissues from GSE3744 (C), GSE14548 (D), and GSE21422 (E) datasets. ↑ and ↓ represent mRNA upregulation and downregulation in breast tumors, respectively. *$P < 0.05$, **$P < 0.01$, ***$P < 0.001$, ****$P < 0.0001$, by two-tailed Student's t test.

(**F**) mRNA expression analysis of the core subunits of SIN3 complex in four molecular subtypes of human breast tumors and normal breast tissues from TCGA breast invasive carcinoma dataset. **$P < 0.01$, ***$P < 0.001$, ****$P < 0.0001$, by one-way ANOVA with Dunnett's test.

(**G**) Validation of anti-SAP30 antibody for immunohistochemical staining in SC and SAP30-KO1 MDA-MB-231 tumors.

(**H**-**L**) Kaplan-Meier survival analysis for patients with breast cancer by log-rank test. Patients were divided by median expression levels of SAP30 mRNA. Data were retrieved from GEO datasets. HR, hazard ratio.

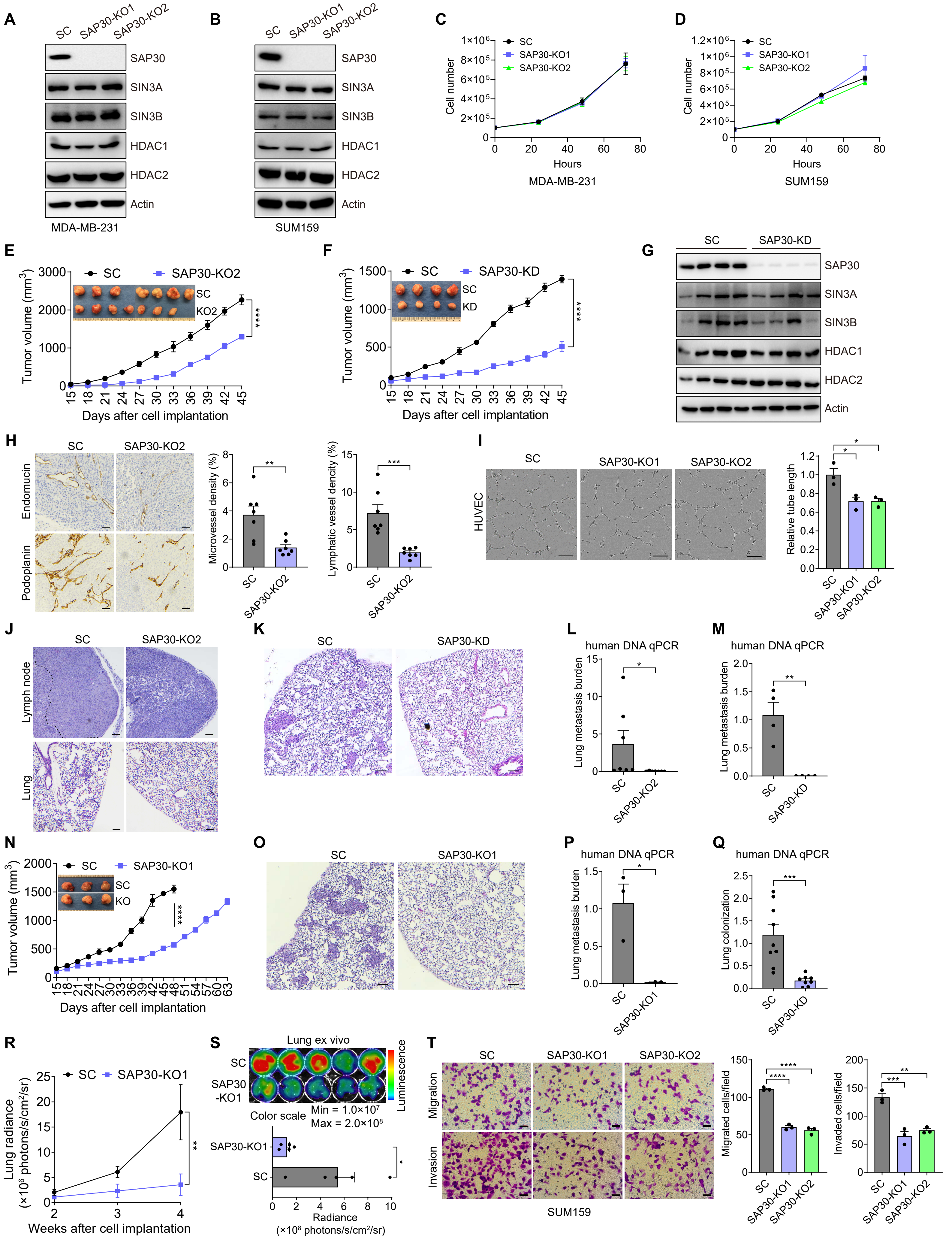

A
SC
SAP30-KO1
SAP30-KO2
SAP30
SIN3A
SIN3B
HDAC1
HDAC2
Actin
MDA-MB-231
B
SUM159
C
Cell number
Hours
MDA-MB-231
D
SUM159
E
Tumor volume (mm3)
Days after cell implantation
F
SAP30-KD
G
H
Endomucin
Podoplanin
Microvessel density (%)
Lymphatic vessel density (%)
I
HUVEC
Relative tube length
J
Lymph node
Lung
K
L
human DNA qPCR
Lung metastasis burden
M
N
O
P
Q
Lung colonization
R
Lung radiance (×10^6 photons/s/cm²/sr)
Weeks after cell implantation
S
Lung ex vivo
Luminescence
Color scale
Min = 1.0×10^7
Max = 2.0×10^8
Radiance (×10^8 photons/s/cm²/sr)
T
Migration
Invasion
Migrated cells/field
Invaded cells/field

Figure S2. SAP30 promotes breast cancer progression in mice.

(**A** and **B**) Immunoblot of indicated proteins in SC and SAP30-KO MDA-MB-231(A) or SUM159 (B) cells. Blots were run in parallel using the same biological samples.

(**C** and **D**) Cell proliferation assay of SC and SAP30-KO MDA-MB-231 (C) or SUM159 (D) cells (mean ± SEM, $n = 3$).

(**E**) Growth of SC and SAP30-KO2 SUM159 tumors in mice (mean ± SEM, $n = 7$). ****$P < 0.0001$, by two-way ANOVA with Šidák's test. The image of tumors harvested at the end time point is shown in the inset.

(**F** and **G**) Growth of SC and SAP30-KD MDA-MB-231 tumors in mice (F, mean ± SEM, $n = 4$). ****$P < 0.0001$, by two-way ANOVA with Šidák's test. The image of tumors harvested at the end time point is shown in the inset. SAP30 protein knockdown in tumors is confirmed by immunoblot assay (G). Blots were run in parallel using the same biological samples.

(**H**) Representative immunohistochemical staining of endomucin and podoplanin in SC and SAP30-KO2 SUM159 tumors (*left*). Endomucin- and podoplanin-positive tumor areas are quantified (*right*, mean ± SEM, $n = 7$). **$P < 0.01$, ***$P < 0.001$, by two-tailed Student's t test. Scale bar, 50 μm.

(**I**) In vitro angiogenesis of HUVECs incubated with conditional media from SC and SAP30-KO1 or -KO2 SUM159 cells (*left*). Total tube length is quantified (*right*, mean ± SEM, $n = 3$). *$P < 0.05$, by one-way ANOVA with Dunnett's test. Scale bar, 100 μm.

(**J**) H&E analysis of lymph node and lung metastasis in mice bearing SC or SAP30-KO2 SUM159 tumors. Scale bar, 100 μm.

(**K**) H&E analysis of lung metastasis in mice bearing SC or SAP30-KD MDA-MB-231 tumors. Scale bar, 100 μm.

(**L** and **M**). qPCR analysis of lung metastasis in mice bearing SC or SAP30-KO2 SUM159 tumors (L, mean ± SEM, $n = 7$) or SC or SAP30-KD MDA-MB-231 tumors (M, mean ± SEM, $n = 4$). *$P < 0.05$, **$P < 0.01$, by two-tailed Student's t test.

(**N**-**P**) Growth (N) and lung metastasis by H&E staining (O) or qPCR assay (P) of SC and SAP30-KO1 MDA-MB-231 tumors in mice (mean ± SEM, $n = 3$). SC and SAP30-KO1 tumor bearing mice were euthanized on day 48 and 63 after cell implantation, respectively. ****$P < 0.0001$, by two-way ANOVA with Šidák's test for growth curve. *$P < 0.05$, by two-tailed Student's t test for qPCR assay. Scale bar, 100 μm. The image of tumors harvested at the end time point is shown in the inset.

(**Q**) Lung colonization of SC and SAP30-KD MDA-MB-231 cells by qPCR assay (mean ± SEM, $n = 9$ for SC and $n = 8$ for SAP30-KD). ***$P < 0.001$, by two-tailed Student's t test.

(**R**) Quantification of bioluminescence imaging of lungs in NSG mice after tail vein injection of SC or SAP30-KO1 MDA-MB-231 cells (mean ± SEM, $n = 5$). **$P < 0.01$, by two-way ANOVA with Šidák's test.

(**S**) Bioluminescence imaging of ex vivo lungs (*top*). Quantification is shown at bottom (mean ± SEM, $n = 5$). *$P < 0.05$, by two-tailed Student's t test.

(**T**) Migration and invasion of SC and SAP30-KO1 or -KO2 SUM159 cells. Representative images from three experiments are shown (*left*) and migrated or invaded cell numbers are quantified (*right*, mean ± SEM, $n = 3$). **$P < 0.01$, ***$P < 0.001$, ****$P < 0.0001$, by one-way ANOVA with Dunnett's test. Scale bar, 50 μm.

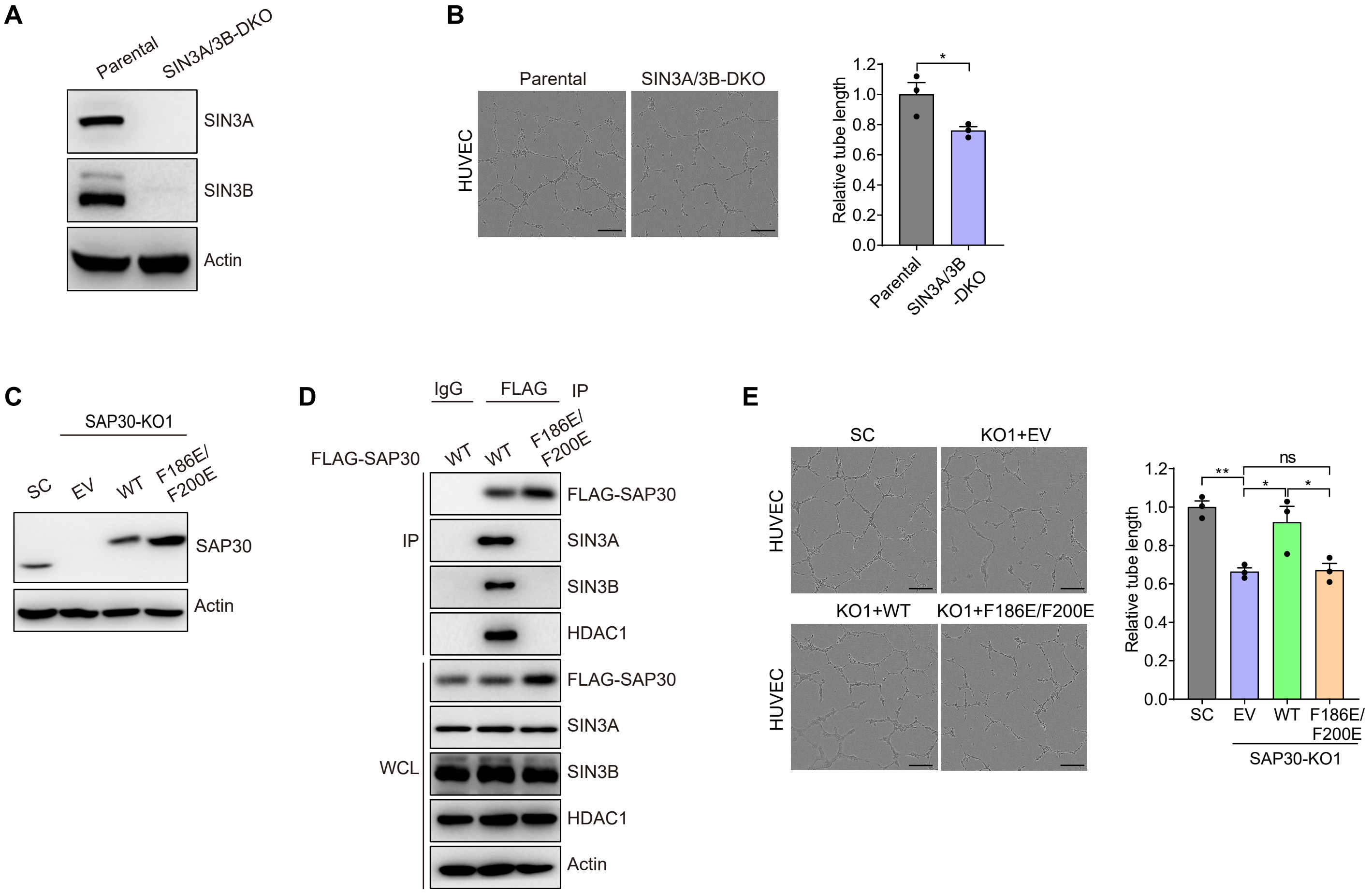

A
Parental
SIN3A/3B-DKO
SIN3A
SIN3B
Actin
B
Parental
SIN3A/3B-DKO
HUVEC
Relative tube length
1.2
1.0
0.8
0.6
0.4
0.2
0.0
*
Parental
SIN3A/3B -DKO
C
SAP30-KO1
SC
EV
WT
F186E/ F200E
SAP30
Actin
D
IgG
FLAG
IP
FLAG-SAP30
WT
WT
F186E/ F200E
IP
WCL
FLAG-SAP30
SIN3A
SIN3B
HDAC1
FLAG-SAP30
SIN3A
SIN3B
HDAC1
Actin
E
SC
KO1+EV
KO1+WT
KO1+F186E/F200E
HUVEC
HUVEC
Relative tube length
**
ns
*
*
SC
EV
WT
F186E/ F200E
SAP30-KO1

Figure S3. SAP30 increases in vitro angiogenesis in a SIN3A/3B-dependent manner.
(**A**) Immunoblot of SIN3A, SIN3B, and actin in parental and SIN3A/3B-DKO MDA-MB-231 cells. Blots were run in parallel using the same biological samples.
(**B**) In vitro angiogenesis of HUVECs incubated with conditional media from parental and SIN3A/3B-DKO MDA-MB-231 cells (*left*). Total tube length is quantified (*right*, mean ± SEM, $n = 3$). $^{*}P < 0.05$, by two-tailed Student's *t* test. Scale bar, 100 μm.
(**C**) Immunoblot of SAP30 and actin in SC, SAP30-KO1, and SAP30-rescue MDA-MB-231 cells. Blots were run in parallel using the same biological samples.
(**D**) Co-IP assay showing that F186E/200E mutation blocks FLAG-SAP30 binding to SIN3 complex in MDA-MB-231 cells ($n = 2$). WCL, whole cell lysate.
(**E**) In vitro angiogenesis of HUVECs incubated with conditional media from SC, SAP30-KO1, and SAP30-rescue MDA-MB-231 cells (*left*). Total tube length is quantified (*right*, mean ± SEM, $n = 3$). $^{*}P < 0.05$, $^{**}P < 0.01$, by one-way ANOVA with Turkey's test. Scale bar, 100 μm.

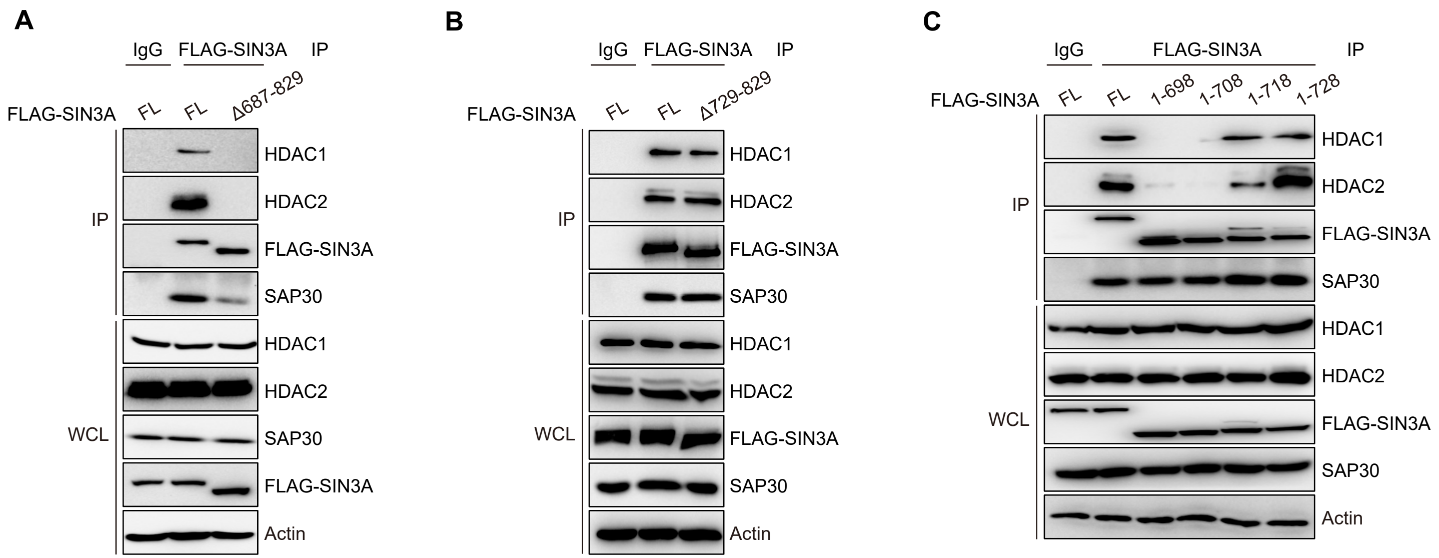
A
IgG
FLAG-SIN3A
IP
FLAG-SIN3A
FL
FL
Δ687-829
IP
HDAC1
HDAC2
FLAG-SIN3A
SAP30
WCL
HDAC1
HDAC2
SAP30
FLAG-SIN3A
Actin
B
IgG
FLAG-SIN3A
IP
FLAG-SIN3A
FL
FL
Δ729-829
IP
HDAC1
HDAC2
FLAG-SIN3A
SAP30
WCL
HDAC1
HDAC2
FLAG-SIN3A
SAP30
Actin
C
IgG
FLAG-SIN3A
IP
FLAG-SIN3A
FL
FL
1-698
1-708
1-718
1-728
IP
HDAC1
HDAC2
FLAG-SIN3A
SAP30
WCL
HDAC1
HDAC2
FLAG-SIN3A
SAP30
Actin

Figure S4. Mapping of SIN3A domain binding to HDAC1/2.
(**A**) Co-IP assay showing that deletion of amino acids 687-829 blocks FLAG-SIN3A binding to HDAC1 and HDAC2 and also impairs SAP30 binding in transfected HEK293T cells ($n = 2$). FL, full-length.
(**B**) Co-IP assay showing that deletion of amino acids 729-829 has no effect on FLAG-SIN3A binding to HDAC1, HDAC2, and SAP30 in transfected HEK293T cells ($n = 2$).
(**C**) Co-IP assay showing interaction of FL FLAG-SIN3A and its N-terminal truncates with HDAC1, HDAC2, and SAP30 in transfected HEK293T cells ($n = 2$).

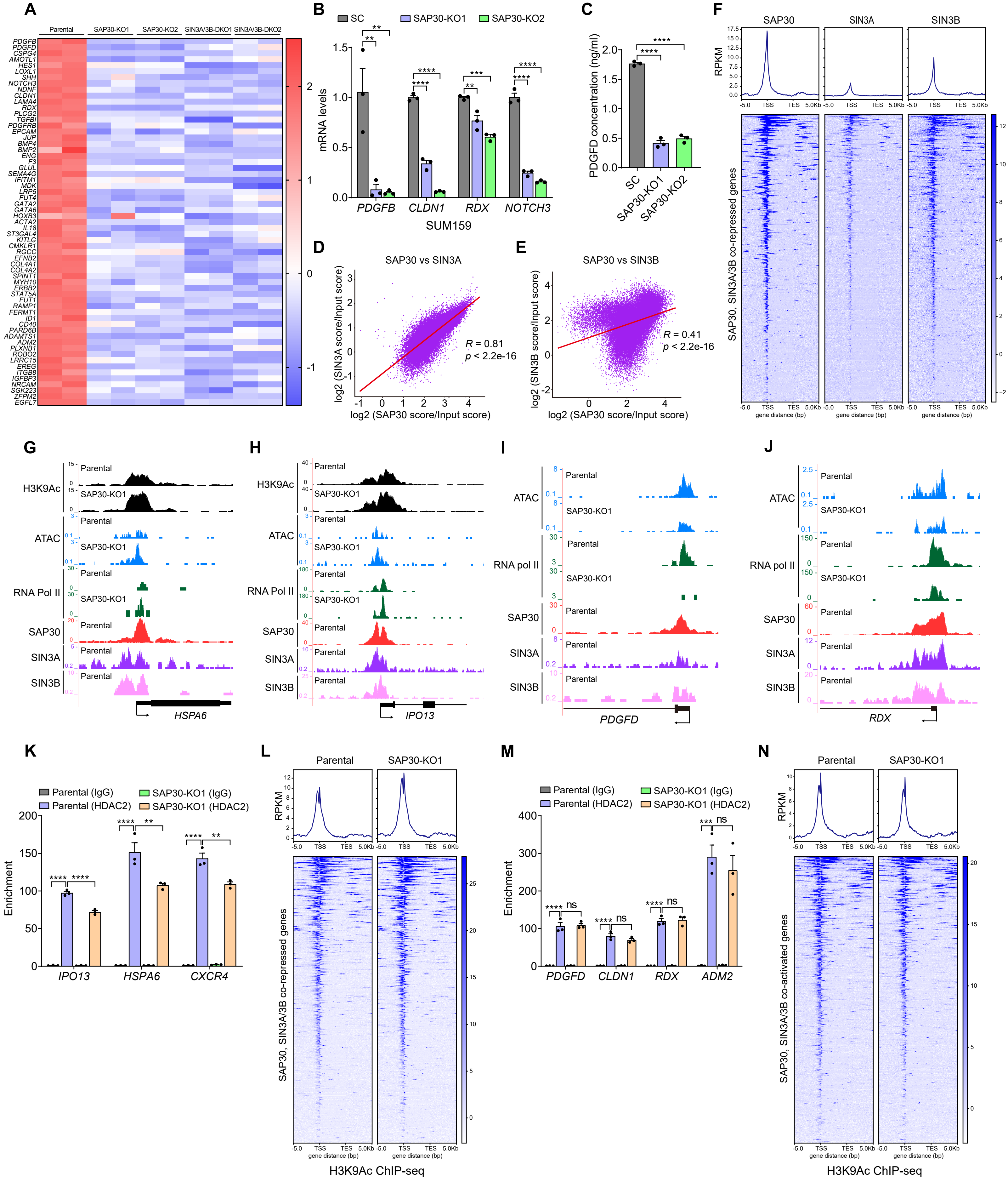

A
Parental
SAP30-KO1
SAP30-KO2
SIN3A/3B-DKO1
SIN3A/3B-DKO2
B
SC
SAP30-KO1
SAP30-KO2
mRNA levels
PDGFB
CLDN1
RDX
NOTCH3
SUM159
C
PDGFD concentration (ng/ml)
SC
SAP30-KO1
SAP30-KO2
D
SAP30 vs SIN3A
log2 (SIN3A score/Input score)
log2 (SAP30 score/Input score)
R = 0.81
p < 2.2e-16
E
SAP30 vs SIN3B
log2 (SIN3B score/Input score)
log2 (SAP30 score/Input score)
R = 0.41
p < 2.2e-16
F
SAP30
SIN3A
SIN3B
RPKM
SAP30, SIN3A/3B co-repressed genes
gene distance (bp)
G
H3K9Ac
ATAC
RNA Pol II
SAP30
SIN3A
SIN3B
Parental
SAP30-KO1
HSPA6
H
IPO13
I
RNA pol II
PDGFD
J
RDX
K
Parental (IgG)
Parental (HDAC2)
SAP30-KO1 (IgG)
SAP30-KO1 (HDAC2)
Enrichment
IPO13
HSPA6
CXCR4
L
SAP30, SIN3A/3B co-repressed genes
H3K9Ac ChIP-seq
M
PDGFD
CLDN1
RDX
ADM2
N
SAP30, SIN3A/3B co-activated genes
H3K9Ac ChIP-seq

Figure S5. SAP30 co-activates genes involved in cell motility, angiogenesis, and lymphangiogenesis in breast cancer cells.

(**A**) Heatmap of angiogenesis and migration genes co-activated by SAP30 and SIN3A/3B in MDA-MB-231 cells (n = 2).
(**B**) RT-qPCR analysis of indicated mRNAs in SC and SAP30-KO SUM159 cells (mean ± SEM, n = 3). *P < 0.05, **P < 0.01, ***P < 0.001, ****P < 0.0001, by one-way ANOVA with Dunnett's test.
(**C**) PDGFD protein levels in media from SC and SAP30-KO MDA-MB-231 cells (mean ± SEM, n=3). ****P < 0.0001, by one-way ANOVA with Dunnett's test.
(**D** and **E**) Scatter plot correlation analysis of ChIP-seq signals between SAP30 and SIN3A (D) or between SAP30 and SIN3B (E) in MDA-MB-231 cells (n = 2).
(**F**) Metagene plot and heatmap of ChIP-seq assay showing occupancies of SAP30, SIN3A, and SIN3B on SAP30, SIN3A/3B co-repressed genes in MDA-MB-231 cells (n = 2). RPKM, reads per kilobase per million mapped reads; TSS, transcription start site; TES, transcription end site.
(**G** and **H**) Genome browser view of ATAC-seq and H3K9Ac, RNA polymerase II, SAP30, SIN3A, SIN3B ChIP-seq peaks of representative SAP30, SIN3A/3B co-repressed genes *HSPA6* (G) and *IPO13* (H) in parental and SAP30-KO1 MDA-MB-231 cells (n = 2).
(**I** and **J**) Genome browser view of ATAC-seq and RNA polymerase II, SAP30, SIN3A, SIN3B ChIP-seq peaks of representative SAP30, SIN3A/3B co-activated genes *PDGFD* (I) and *RDX* (J) in parental and SAP30-KO1 MDA-MB-231 cells (n = 2).
(**K** and **M**) ChIP-qPCR assay showing relative HDAC2 occupancy on representative SAP30, SIN3A/3B co-repressed genes (K) and co-activated genes (M) in parental and SAP30-KO1 MDA-MB-231 cells (mean ± SEM, n = 3). **P < 0.01, ***P < 0.001, ****P < 0.0001, by two-way ANOVA with Turkey's test.
(**L** and **N**) Metagene plot and heatmap of ChIP-seq assay showing H3K9Ac occupancy on SAP30, SIN3A/3B co-repressed genes (L) and co-activated genes (N) in parental and SAP30-KO1 MDA-MB-231 cells (n = 2).

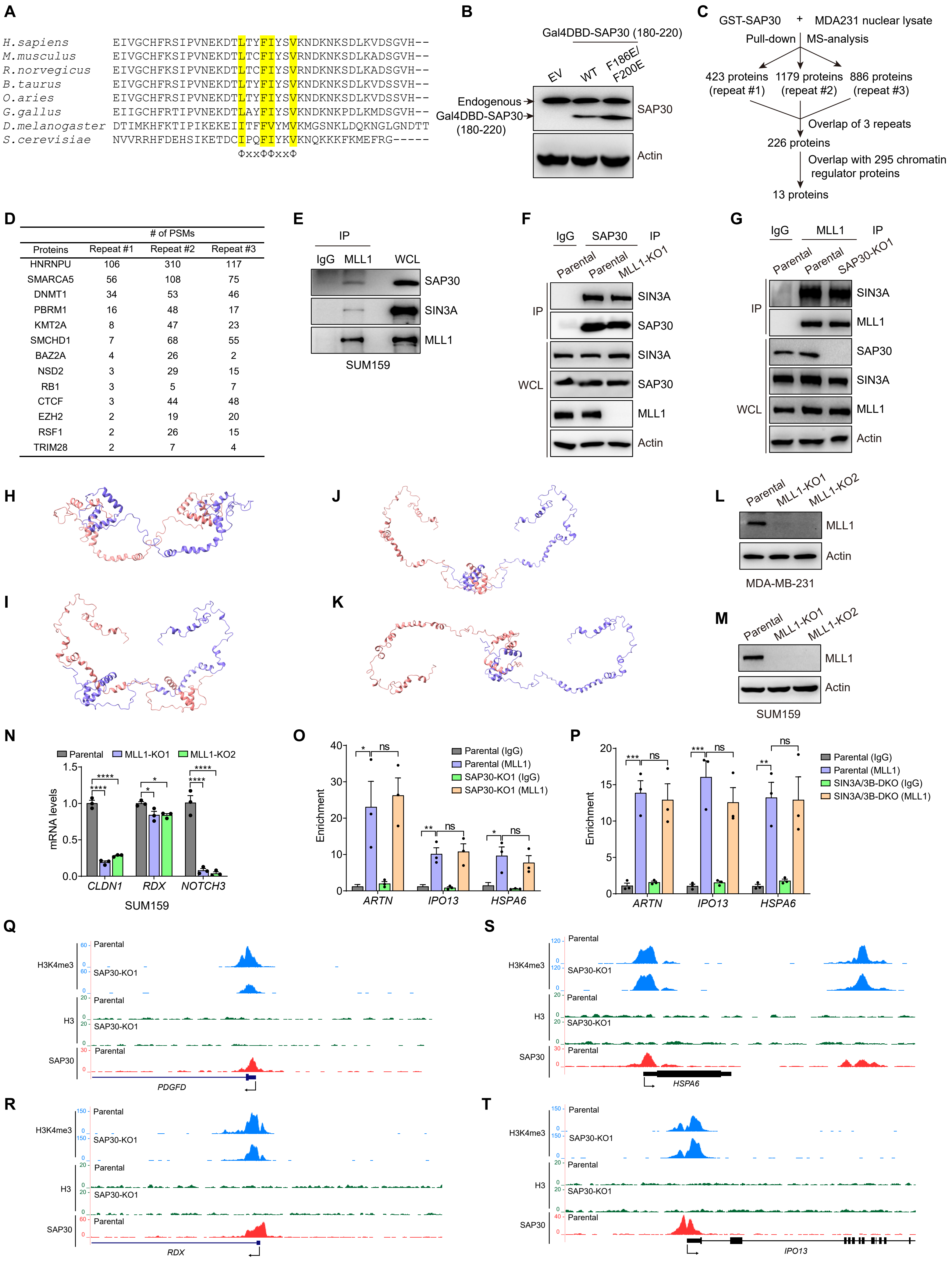

Proteins	Repeat #1	Repeat #2	Repeat #3
	# of PSMs		
HNRNPU	106	310	117
SMARCA5	56	108	75
DNMT1	34	53	46
PBRM1	16	48	17
KMT2A	8	47	23
SMCHD1	7	68	55
BAZ2A	4	26	2
NSD2	3	29	15
RB1	3	5	7
CTCF	3	44	48
EZH2	2	19	20
RSF1	2	26	15
TRIM28	2	7	4

Figure S6. MLL1 is required for SAP30 coactivator function in breast cancer cells.

(**A**) Protein sequence alignment of the conserved motifs ϕ-×-×-ϕ-ϕ and ϕ-ϕ-×-×-ϕ within the transactivation domain of SAP30 across different species. Hydrophobic residues (ϕ) in the motifs are highlighted in yellow.

(**B**) Immunoblot of Gal4DBD-WT SAP30 (180-220 aa) and Gal4DBD-F186E/F200E SAP30 (180-220 aa) in transfected HEK293T cells.

(**C**) Scheme of GST-SAP30 pulldown followed by mass spectrometry (MS) assay to identify SAP30-binding proteins.

(**D**) A list of epigenetic regulators interacting with SAP30. #PSM, Number of Peptide Spectrum Matches.

(**E**) Co-IP assay showing that MLL1 interacts with SAP30 and SIN3A in SUM159 cells ($n = 2$).

(**F**) Co-IP assay showing that MLL1 KO has no effect on SAP30-SIN3A interaction in MDA-MB-231 cells ($n = 2$).

(**G**) Co-IP assay showing that SAP30 KO has no effect on MLL1-SIN3A interaction in MDA-MB-231 cells ($n = 2$).

(**H**-**K**) Top four structural modeling of human SAP30 homodimer.

(**L** and **M**) Immunoblot of indicated proteins in parental and MLL1-KO1 or -KO-2 MDA-MB-231 (L) and SUM159 (M) cells.

(**N**) RT-qPCR analysis of indicated mRNAs in parental and MLL1-KO SUM159 cells (mean ± SEM, $n = 3$). *$P < 0.05$, ****$P < 0.0001$, by one-way ANOVA with Dunnett's test.

(**O** and **P**) ChIP-qPCR assay showing relative MLL1 occupancy on representative SAP30, SIN3A/3B co-repressed genes in parental, SAP30-KO1 (O), SIN3A/3B-DKO (P) MDA-MB-231 cells (mean ± SEM, $n = 3$). *$P < 0.05$, **$P < 0.01$, ***$P < 0.001$, by two-way ANOVA with Turkey's test.

(**Q**-**T**) Genome browser view of H3K4me3, histone H3 and SAP30 ChIP-seq peaks of representative SAP30, SIN3A/3B, MLL1 co-activated genes *PDGFD* (Q), *RDX* (R) and representative SAP30, SIN3A/3B co-repressed genes *HSPA6* (S), *IPO13* (T) in parental and SAP30-KO1 MDA-MB-231 cells ($n = 2$).

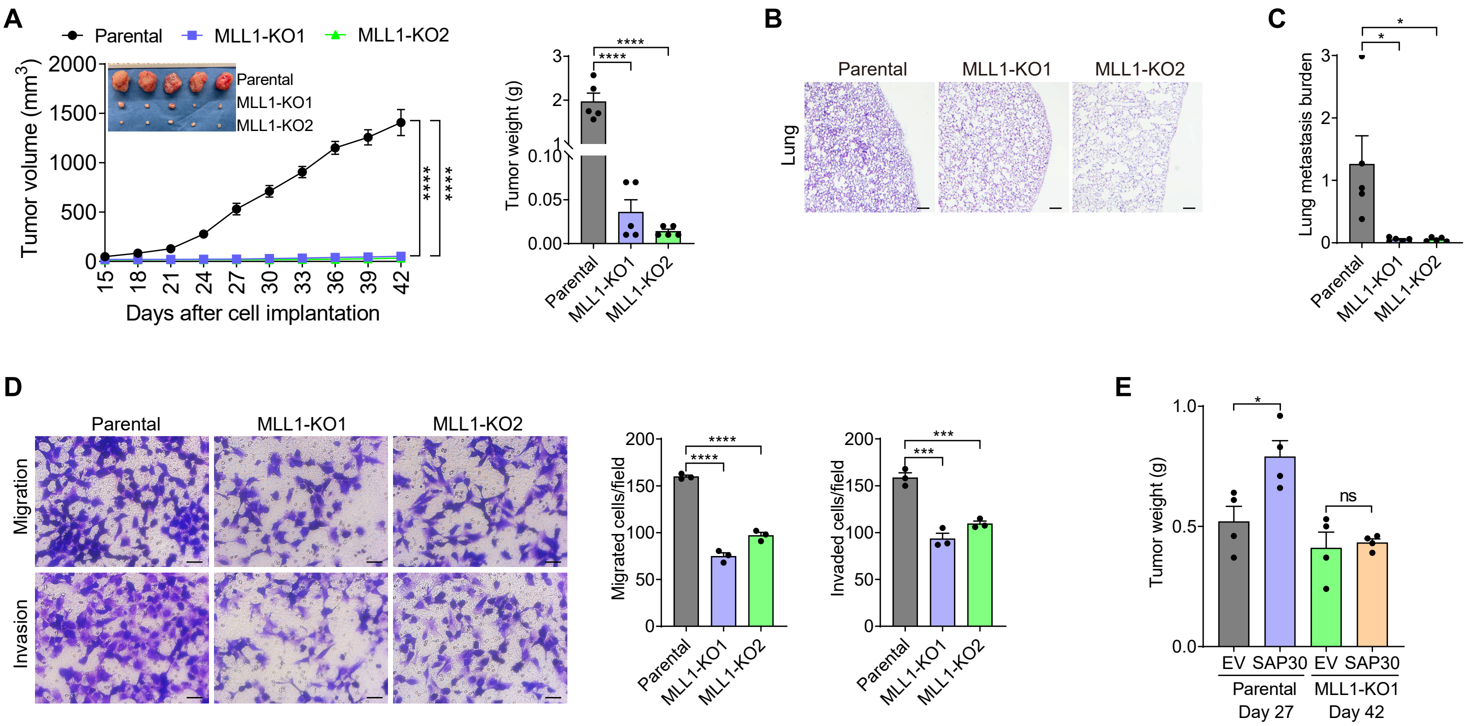

A
Parental
MLL1-KO1
MLL1-KO2
Tumor volume (mm3)
2000
1500
1000
500
0
15
18
21
24
27
30
33
36
39
42
Days after cell implantation
****
****
Tumor weight (g)
3
2
1
0.10
0.05
0.00
B
Parental
MLL1-KO1
MLL1-KO2
Lung
C
Lung metastasis burden
*
*
D
Parental
MLL1-KO1
MLL1-KO2
Migration
Invasion
Migrated cells/field
Invaded cells/field
200
150
100
50
0
***
E
Tumor weight (g)
1.0
0.5
0.0
ns
EV
SAP30
EV
SAP30
Parental
Day 27
MLL1-KO1
Day 42

Figure S7. MLL1 is necessary for SAP30-mediated breast cancer progression.

(**A**) Growth of parental and MLL1-KO1 or -KO2 SUM159 tumors in mice (mean ± SEM, $n = 5$). After harvesting, tumors are imaged and weighed. ****$P < 0.0001$, by two-way ANOVA with Dunnett's test (*left*) or one-way ANOVA with Dunnett's test (*right*).

(**B** and **C**) Lung metastasis in mice bearing parental and MLL1-KO1 or -KO2 SUM159 tumors by H&E staining (B) and qPCR assay (C, mean ± SEM). $n = 5$. *$P < 0.05$, by one-way ANOVA with Dunnett's test. Scale bar, 100 µm.

(**D**) Migration and invasion of parental and MLL1-KO1 or -KO2 SUM159 cells (*left*). Migrated or invaded cell numbers are quantified (*right*, mean ± SEM). $n = 3$. ***$P < 0.001$, ****$P < 0.0001$, by one-way ANOVA with Dunnett's test. Scale bar, 50 µm.

(**E**) Weight of SAP30 overexpressed parental and MLL1-KO1 MDA-MB-231 tumors in mice (mean ± SEM, $n = 4$). Parental+EV/SAP30 and MLL1-KO1+EV/SAP30 tumor bearing mice are euthanized on day 27 and 42 after cell implantation, respectively. *$P < 0.05$ by two-tailed Student's t test.
